# Individual perspectives and mental maps of working conditions and intention to stay of physicians in academic medicine

**DOI:** 10.3389/fpsyg.2023.1106501

**Published:** 2023-05-12

**Authors:** Joachim Hasebrook, Juliane Hecke, Thomas Volkert, Maren Singer, Juergen Hinkelmann, Leonie Michalak, Klaus Hahnenkamp

**Affiliations:** ^1^ZEB Business School, Steinbeis University, Berlin, Germany; ^2^Department of Anesthesiology, University Hospital Muenster, Münster, Germany; ^3^Department of Anesthesiology, Lukas Hospital Dortmund, Dortmund, Germany; ^4^CURACON GmbH, Münster, Germany; ^5^Department of Anesthesiology, University Medicine Greifswald, Greifswald, Germany

**Keywords:** job satisfaction, physicians, turnover intention, teamwork, skills shortage, interview study, repertory grids

## Abstract

**Introduction:**

Job satisfaction has a strong impact on the intention to stay which is an important aspect to counter skills shortage in academic medicine. The purpose of the three studies reported here is to find out what specific factors are relevant for the intention to stay and turnover intention of physicians in academic medicine –and what measures might have a positive impact on employee retention.

**Methods:**

In an interview study combining qualitative and quantitative methods, we investigated how the individual mental representation of working conditions influences job satisfaction and its impact on the intention to stay. In total, 178 physicians from German university hospitals, residents, and physicians, in 15 departments of anesthesiology were interviewed and surveyed. In a first study, chief physicians participated in interviews about job satisfaction in academic hospitals. Answers were segmented into statements, ordered by topics, and rated according to their valence. In a second study, assistant physicians during and after their training period talked about strengths, weaknesses, and potential improvements of working conditions. Answers were segmented, ordered, rated, and used to develop a “satisfaction scale.” In a third study, physicians participated in a computer-led repertory grid procedure composing ‘mental maps’ of job satisfaction factors, filled in the job satisfaction scale and rated if they would recommend work and training in their clinic as well as their intention to stay.

**Results:**

Comparing the interview results with recommendation rates and intention to stay show that high workload and poor career perspectives are linked to a negative attitude. A positive attitude towards work environment and high intention to stay is based on sufficient personnel and technical capacities, reliable duty scheduling and fair salaries. The third study using repertory grids showed that the perception of current teamwork and future developments concerning work environment were the main aspects to improve job satisfaction and the intention to stay.

**Discussion:**

The results of the interview studies were used to develop an array of adaptive improvement measure. The results support prior findings that job dissatisfaction is mostly based on generally known “hygiene factors” and whereas job satisfaction is due to individual aspects.

## 1. Introduction

### 1.1. Determinants of job satisfaction and intention to stay

Job satisfaction received attention from various scientific fields for decades and remains a focal point of research concerning management practice (Zhu, 2017). Measuring job satisfaction serves as an indicator for employees’ performance in terms of quality (Judge et al., 2020), productivity and commitment (Spector, 1997; Coetzee and Stoltz, 2015) as well as for turnover risks (Halter et al., 2017; Radford and Meissner, 2017; Yarbrough et al., 2017; De Simone et al., 2018; Judge et al., 2020; Koch et al., 2020; Labrague, 2020; Nikkhah-Farkhani and Piotrowski, 2020; Sillero-Sillero and Zabalegui, 2020). Additionally, research was able to show that satisfied employees are more likely to advocate for the organization (Judge et al., 2020). Concerning healthcare, job satisfaction is of paramount importance, because it is directly linked to patients’ satisfaction (Haas et al., 2000), patient safety (Aiken et al., 2002), and outcome (Katz, 1999). In consequence, physicians’ job satisfaction has an impact on national health outcomes (Oh et al., 2019).

A fundamental definition of job satisfaction refers to the way people feel about their job and whether they like or dislike it (Spector, 1997)—a rather simple definition frequently used and built upon (Moorman, 1993; Ajamieh et al., 1996; Armstrong, 2006; Giménez-Espert et al., 2020). If the employees have positive feelings and attitudes toward their jobs (Ajamieh et al., 1996; Giménez-Espert et al., 2020) and are enthusiastic and happy with their work (Kaliski, 2007), they report being satisfied with their job. Other research expands job satisfaction by adding the personal feeling of achievement (Statt, 2004; Mullins, 2005; Kaliski, 2007). Receiving rewards–equally intrinsically and extrinsically—are associated with job satisfaction, if they are perceived as rightfully received (Mullins, 2005; Rama-Maceiras et al., 2012; Dall’Ora et al., 2020). Furthermore, Davis and Newstrom (1989) state that job satisfaction deals with meeting or exceeding the employees’ expectations of the job. Moorman (1993) differentiates between affective and cognitive perspectives on job satisfaction. Cognitive satisfaction is a logical and rational evaluation of working conditions, which are crucial factors to be examined in order to understand job satisfaction (Rama-Maceiras et al., 2012; Heponiemi et al., 2014; Adriaenssens et al., 2015; Dall’Ora et al., 2015; Barken et al., 2018; Kao et al., 2018; Kim and Yi, 2019; Pishgooie et al., 2019; Tawfik et al., 2019; Bautista et al., 2020; Labrague et al., 2020; Rodríguez-García et al., 2021a,b).

There is evidence for various working conditions influencing job satisfaction. Among them are: Relatively high and fair salary (Kao et al., 2018), opportunities for promotion and social aspects of work (Pocztowski, 2003; Armstrong, 2006; Sypniewska, 2014), ethical and transformational leadership, (Kim and Yi, 2019; Pishgooie et al., 2019; Tawfik et al., 2019; Labrague et al., 2020; McKenna and Jeske, 2021) as well as sustainable relationships with supervisors (Rama-Maceiras et al., 2012; Dall’Ora et al., 2020), and co-workers (Kim and Yi, 2019; Dall’Ora et al., 2020). A good perceived atmosphere at work (Sypniewska, 2014; Tawfik et al., 2019) also results in high job satisfaction. Conversely, high workload and stress factors–like time pressure–have a negative impact on job satisfaction (Jermsittiparsert et al., 2021). Herzberg (1965) categorized working conditions influencing job satisfaction into external and internal factors: External or hygiene factors comprise wages, safety, and supervisors. Internal factors or motivators refer to higher needs, such as recognition by others, work performance, development, and accountability.

### 1.2. Components of job satisfaction

Martinussen et al. (2020) found that 21% of all hospital physicians have the intention to leave their current job. Furthermore, over 20% were indecisive. The COVID-19 pandemic has deepened the attrition crises (Alrawashdeh et al., 2021) and increased the intention to leave especially among physicians, who tend to be more mobile than nurses and find better paying jobs more easily (Jackson et al., 2018; Johna, 2018). However, most research results indicate that similar factors account for job satisfaction of physicians and nurses. The social climate was a factor favoring the nurses’ and physicians’ intentions to stay with their employer (Heponiemi et al., 2019; Martinussen et al., 2020; Nikkhah-Farkhani and Piotrowski, 2020). Leadership style had a decisive influence on physicians’ intentions to stay or to leave (Stagnitti et al., 2006; Suliman, 2009; Halter et al., 2017; Fontes et al., 2019; Pishgooie et al., 2019; Labrague et al., 2020; Lee and Jang, 2020; Magbity et al., 2020; Martinussen et al., 2020). Other factors pushing physicians’ and nurses’ intentions toward leaving their job were: Negative feelings when experiencing discrimination (Heponiemi et al., 2019), bullying (Adriaenssens et al., 2015; Edmonson and Zelonka, 2019; Park and Choi, 2019; Favaro et al., 2021), conflict with peers (Zaheer et al., 2019; Bautista et al., 2020; Lee and Kim, 2020), high workloads (Perkins et al., 2007; Bautista et al., 2020; Lee and Kim, 2020), understaffing (Sasso et al., 2019), emotional exhaustion (Hoonakker et al., 2013; Vandenbroeck et al., 2017; Sasso et al., 2019), long working shifts (Dall’Ora et al., 2015; Arslan Yürümezoğlu et al., 2019), and high stress (Hoonakker et al., 2013; Halter et al., 2017; Lee and Jang, 2020; Somville et al., 2020; Chen et al., 2021). Several factors affecting job satisfaction also influence physicians’ and nurses’ intention to stay: Fair pay (Kao et al., 2018), freedom to do the job (Barken et al., 2018), job autonomy (Stagnitti et al., 2006; Barken et al., 2018), and recognition (Yoder, 1995; Adriaenssens et al., 2015; Hämmig, 2018). Also, work-family conflicts or work-life imbalances (Hämmig, 2018; HakemZadeh et al., 2020; Nikkhah-Farkhani and Piotrowski, 2020) and perceived poor career perspectives (Yoder, 1995; Perkins et al., 2007) were found to be factors leading physicians and nurses to quit their jobs.

Although physicians and nurses do not differ in their perspective on work conditions in most aspects (Domagała et al., 2018; Heponiemi et al., 2019), some aspects are distinctive: Fair pay (Kao et al., 2018; Oh et al., 2019), management and leadership (Nassab, 2008; Martinussen et al., 2020), career development through specialization (Leigh et al., 2002) or social support, and social climate (Stagnitti et al., 2006; Adriaenssens et al., 2015; Dall’Ora et al., 2020) as well as good relationships with other colleagues (Stoddard et al., 2001; Sibbald et al., 2003; Domagała et al., 2018; Oh et al., 2019) and adequate communication among peers (Sibbald et al., 2003) are influencing physicians’ job satisfaction. Physicians value good relations with their patients as they tend to attest to higher job satisfaction if they have adequate time to spend with patients, and if they are able to maintain relationships with them (Stoddard et al., 2001; Oh et al., 2019). Job satisfaction is enhanced by a high level of professional autonomy and freedom, which is perceived as that and considered to be guaranteed (Stagnitti et al., 2006; Rama-Maceiras et al., 2012; Barken et al., 2018; Oh et al., 2019; Dall’Ora et al., 2020). Social status and reputation is an influencing factor, especially for physicians’ job satisfaction (Oh et al., 2019). In general, a good working environment over all, and flexible work conditions in particular have a positive effect on satisfaction (Stagnitti et al., 2006). In contrast, long and swing shifts (Grainger et al., 1995), high workload (Dall’Ora et al., 2015; Bautista et al., 2020), high job demands (Adriaenssens et al., 2015; Dall’Ora et al., 2020), low job control (Rama-Maceiras et al., 2012; Adriaenssens et al., 2015; Dall’Ora et al., 2020), and long working hours (Leigh et al., 2002; Dall’Ora et al., 2015; Oh et al., 2019; Dall’Ora et al., 2020) lower job satisfaction leading to job dissatisfaction as well as negative feelings and attitudes toward the job (Armstrong, 2006).

### 1.3. Outcomes of job (dis)satisfaction

Dissatisfaction and negative attitudes have a number of undesirable consequences: Decreased performances and loyalty, increased absenteeism (Aziri, 2011), and higher risk of employee turnover (Halter et al., 2017; Radford and Meissner, 2017; Yarbrough et al., 2017; De Simone et al., 2018; Judge et al., 2020; Koch et al., 2020; Labrague, 2020; Nikkhah-Farkhani and Piotrowski, 2020; Sillero-Sillero and Zabalegui, 2020). Patients’ safety may be affected: High workload, capacity shortages, and dissatisfaction of physicians have significant impacts on performance and patients’ safety (Aiken et al., 2002; Catalá-López, 2009). This may also lead to financial losses (Weninger Henderson, 2020). Job satisfaction of caregivers is directly linked to their intention to stay (Radford and Meissner, 2017; Yarbrough et al., 2017; Nikkhah-Farkhani and Piotrowski, 2020; Rodríguez-García et al., 2021a,b), whereas dissatisfaction was found to be a driving force for turnover intention (Hoonakker et al., 2013; Halter et al., 2017; De Simone et al., 2018; Koch et al., 2020; Labrague, 2020; Sillero-Sillero and Zabalegui, 2020). Turnover intention is not the opposite of intention to stay (Nanncarrow et al., 2014) because dissatisfaction and intentions to leave is not drawn from the same working conditions as satisfaction and intentions to stay (Perkins et al., 2007). For instance, physicians might want to stay with the employer because of the great communication between colleagues while simultaneously they may have the intention to leave because they find better career opportunities elsewhere.

Considering increasing skills shortages and endangered patients’ safety, it is crucial for hospitals to evaluate employees’ job satisfaction as well as their intentions to leave or stay via active retention management (Gerson, 2002). Employee retention helps to ensure competence continuity in critical care settings demanding high expertise and experience of caregivers (Shewchuk et al., 2006; Hahnenkamp et al., 2018). Experienced physicians are sought after within the labor market and often feel more attached to their profession than their employer (Martinussen et al., 2020). Therefore, employers have to provide convincing arguments for physicians to stay with them (Mano-Negrin and Kirschenbaum, 1999). Those arguments must target the aspects of high job satisfaction and low intention to stay.

### 1.4. Project background and research questions

The purpose of the three studies reported here is to find out what specific factors are relevant for the intention to stay and turnover intention of physicians in academic medicine –and what measures might have a positive impact on employee retention. This is not as easy as it seems considering the multitudinous research results. Reviews of this research (Halter et al., 2017; Fontes et al., 2019) show that most data are derived from standardized surveys based on certain models such as Leader-Membership-Exchange (LMX, Pishgooie et al., 2019; Labrague et al., 2020; McKenna and Jeske, 2021) or Job Demands-Resources model (JDR, Hoonakker et al., 2013). Moreover, the results refer to various perspectives, such as career development (Nassab, 2008), economic issues (Weninger Henderson, 2020), or employer attractiveness (Rodríguez-García et al., 2021a,b). In addition, a variety of work conditions, such as fair pay (Kao et al., 2018) shift work (Dall’Ora et al., 2015), and personal perceptions –e.g., concerning recognition (Adriaenssens et al., 2015) and autonomy (Barken et al., 2018)—as well as cohort effects (Gordon, 2017) play an important role to determine job satisfaction and satisfaction as well as its impact on intention to stay and turnover. In summary, despite the overwhelming amount of research data, there is a lack of individual perspectives, which are addressed in some case studies (Liedtka et al., 1998) or through participatory observation (Morrison and Korol, 2014). We want to bridge the gap between quantitative, model-based research based on standardized surveys and qualitative approaches and bring back the individual perspective to the design of retention programs in hospitals (Shewchuk et al., 2006). We combined quantitative and qualitative research in two different ways: (1) Indirect by combining a qualitative research line with interviews and a quantitative line with surveys and (2) direct by applying Repertory Grids as a method, which combines qualitative and quantitative elements (White, 1996).

Interviews and surveys carried out within the “FacharztPlus” project (further referred to as “PhysicianPlus”), a project financially supported by the German Ministry of Education and Research (BMBF) and aiming toward finding measures in order to retain physicians in hospitals (Hasebrook et al., 2016a). Physicians from German university hospitals, residents and physicians, in 15 departments of anesthesiology were interviewed and surveyed (Hinkelmann et al., 2017). The results were used to evaluate measures to increase job satisfaction in other industries which have to deal with shift work, a need for an adaptive and at least partly highly qualified workforce, such as professional services, harbor and airport logistics. Positively evaluated measures were adapted and tested in the participating hospitals (Hahnenkamp and Hasebrook, 2022).

The research within the PhysicianPlus project was guided by four research questions:

(1). How are negative and positive statements in individual interviews structured and how they are interrelated?
(2). How are individual negative statements (weaknesses) and positive statements (strengths) connected to the individual valuation of work and training quality as well as the intention to stay?
(3). How are individual statements and valuations affected by cohort effects, such as work experience and career stage?
(4). How do “mental maps” summarizing individual positive and negative statements of the different cohorts differ with respect to work, training, and intention to stay?

Answers to these research questions should help to reflect on practical implications and to develop and test measures to improve intention to stay and reduce turnover intention of highly qualified staff in hospitals.

## 2. Materials and methods

### 2.1. Design

**FIGURE 1:**
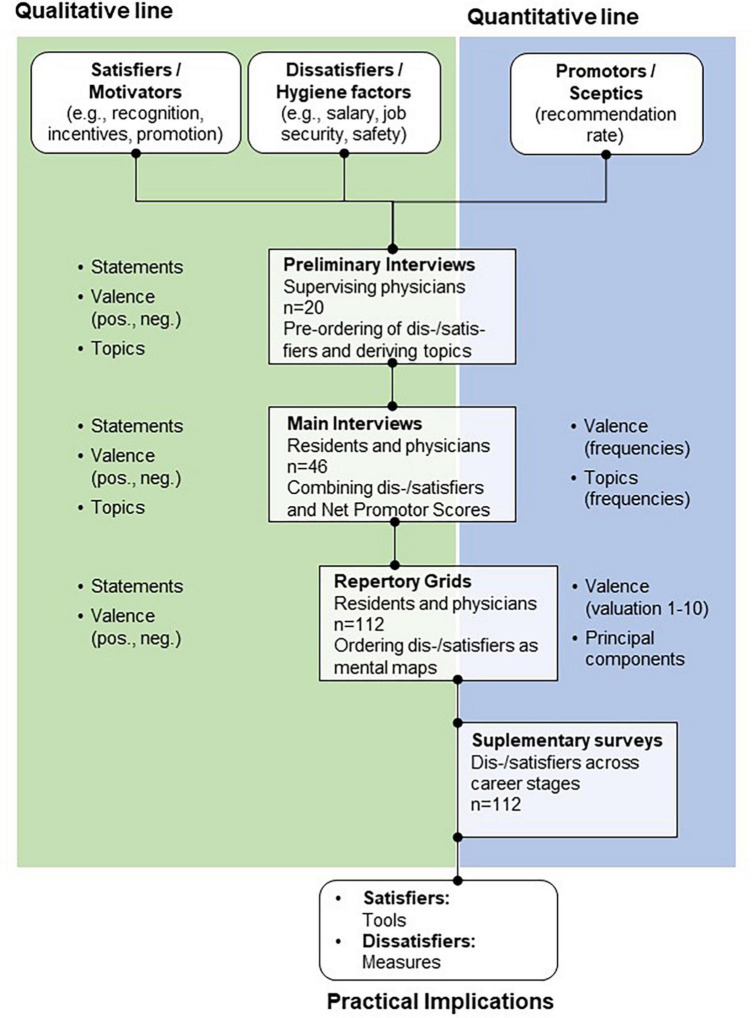
Swimlane of complementary qualitative and quantitative mixed-method design used in the interview study.

We used qualitative and quantitative methods in a convergent parallel mixed-method design (Alrawashdeh et al., 2021). Our mixed-method approach integrates qualitative and quantitative methods at multiple steps of research (Teddlie and Yu, 2007; Fetters et al., 2013; Moseholm and Fetters, 2017; Alrawashdeh et al., 2021; cf. Figure 1).

#### 2.1.1. Interviews

Individuals participated in semi-structured interviews giving them the freedom to mention all aspects concerning their work in the hospital, which were later rated as “negative” or “positive” and related to topics derived from the interviews, such as “vacation scheduling” or “onboarding processes for new employees”.

#### 2.1.2. Surveys

Three different types of surveys were used: (a) The Net Promoter Score (NPS), (b) a polarity satisfaction scale derived from the interview results, and (c) Repertory (or Kelly) Grids.

(a) Net Promoter Score (NPS): The NPS was originally developed to measure customer loyalty (Reichheld, 2003) but is also applied in hospitals (Melissant et al., 2018; Sieja et al., 2019; Garcia-Huidobro et al., 2020; Bosch et al., 2021) especially to measure satisfaction with the work environment (Legerstee, 2013; Vochin et al., 2020). The NPS measures the willingness of a person (a customer or an employee) to recommend a company, a product or working conditions to relevant others, such as family members, friends, or colleagues on a scale from 0 (very unlikely) to 10 (very likely). This ensures a thorough self-evaluation whether to recommend a company or not (De Haan et al., 2015). Only responses 9 and 10 are seen as active promotors, whereas responses from 0 to 6 are valued as “skeptical,” because only a very positive evaluation leads to active, promoting behavior (Eklof et al., 2020).

(b) Polarity satisfaction scale: Based on the results of the semi-structured interviews a polarity scale describing dissatisfiers on the one side and satisfiers on the other was developed reflecting the most distinctive interview mentions concerning satisfactory and dissatisfactory work aspects.

(c) Repertory Grids: Based on Kelly’s personal construct theory (Blowers and O’Connor, 1995) Kelly Grids (or Repertory Grids) measure the valence of predefined elements in terms of personal constructs. Repertory Grids combine both qualitative and quantitative methods and eliminate interviewer bias, because participants essentially create their own questionnaire (Winter, 2003). They gathered data not only on factors required for success, but the relative importance of each factor to the concept of successful practice (White, 1996). It is a methodology commonly employed in Job Analysis i.e., describing jobs and the attributes required to perform them, and provides the workers’ perspective (Hill et al., 2016; Hamad et al., 2017; Burke, 2022). The Repertory Grids were analyzed, visualized as “mental maps” of job satisfaction and dissatisfaction aspects, and they were related to NPS expressing satisfaction with working conditions, training, and intention to stay.

### 2.2. Material and procedure in general

In the first study, semi-structured individual interviews were used as a tool for data collection (Supplementary Appendix 1). In a first study chief physicians were interviewed about their experiences concerning keeping or leaving jobs in academic hospitals. The answers were protocolled, segmented into statements and these statements were aggregated with the help of a hierarchical cluster analysis (Husson et al., 2010).

In a second study residents talked about strengths, weaknesses, and potential improvements of working conditions in their hospital within the framework of open, semi-structured interviews. Answers were recorded, segmented into statements, and assigned to the cluster items obtained from the first study. Additionally, all physicians rated their recommendation concerning work environment, career, education, and intention to stay applying the NPS procedure.

In a third study, residents from four university hospitals participated in a brief interview including a computer-led Repertory Grid procedure. We asked the participants to respond to eight elements: 1. “nurses” and 2. “physicians” (their profession), 3. “clinic today” and 4. “clinic in 5 years’ time” (present and future or their workplace), 5. “hospital today” and 6. “hospital in 5 years’ time” (present and future of the institution) as well as 7. “clinic management,” and 8. “hospital management” (their direct management and general management of the institution). In a first run of the computer-led survey, participants were asked to name a typical feature for each element, e.g., “nurses—team cohesion” or “physicians—high expertise.” They were also asked to state whether this feature is positive or negative, e.g., “team cohesion” = positive, and describe the opposite, e.g., “team conflict” = negative. In a second run, the participants responded to pairs of elements, such as “nurses—physicians.” They were given their statements from the first run as a scale from 1 = negative to 10 = positive and evaluated each element on this individual scale, e.g., “Nurses—1 = team conflict to 10 = team cohesion” and “Physicians—1 = team conflict to 10 = team cohesion.” As a result, all elements were individually described by each participant with its most relevant features, and the valence of all elements was evaluated on scale from negative = 1 to positive = 10.

## 3. Results

### 3.1. Study 1: deriving a rating procedure and topic list

#### 3.1.1. Design

In a semi-structured interview chief physicians (residents) were asked to talk about strengths, weaknesses and ideas for improvements concerning clinical work, team culture and cooperation, management and leadership, training, and development as well as prospects of the project PhysicianPlus itself (see Supplementary Appendix 1.1; original version in Supplementary Material 1). All interview phrases were written down and split up into single statements (see Supplementary Material 3 for a sample protocol). These statements were evaluated by independent raters not participating in the interview process, according to valence (positive, negative) and topic affiliation of each statement. The topics were in two steps: Automatic ordering of phrases using cluster analyses, and further refinement during the rating process. The results of this rating procedure form the basis of the main interview study.

#### 3.1.2. Participants

In summary, n = 20 residents participated in the interviews, n = 4 female and n = 16 male persons. In average, they held their positions for 4.4 years and worked for the hospital in average for 13.6 years.

#### 3.1.3. Method and procedure

Interviews lasted about 45 to 180 min (mean 1.5 h) and were recorded in a written protocol (example see Supplementary Material 3). The protocols were checked by the interviewees, who gave their written consent that the protocols may be used in this research. Protocols were anonymized and coded in a codebook transferring original phrases into abbreviated statements. These were rated according to valence (positive vs. negative) and topic affiliation. Raters used a topic list automatically generated through a cluster analysis of all statements collected in all interviews and refined it step by step during the rating of all interviews. A brief sample of the codebook is shown in Table 1, the full codebook is provided as Supplementary Material 4).

**TABLE 1:** Excerpt from the codebook to derive short statements from original interview phrases and aggregate them to topics (translated from the German original).

Original phrase	Short statement	Sentiment	Topic
Size and competence in patient care, proud to work “state of the art”	Medical expertise (state-of-the-art, modern), competence	Positive	Quality of training
Differences in position are sometimes played out (let the doctor “run up,” let the nurse “fidget”)	Playing out differences in position	Negative	Cooperation with nurses
Specialist often is a “motivator” and “explainer” (e.g., that waiting times and short usage times of expensive devices cause costs)	Physician as mentor/supervisor	Unclear valence	Quality of supervision

All examples are taken from the interview with subject #1; the full interview is documented in a Supplementary material as well as the coding book with respect to 46 subjects participating in the main interview study.

#### 3.1.4. Results

In total, physicians generated 560 positive or negative statements about their work in the clinic. The aggregation of all statements resulted in 20 high level topics. Most items referred to quality of training (14.3%) and workforce planning (12.0%) followed by cooperation and culture (10.2%). Results of the aggregation are shown in Table 2.

**TABLE 2:** Frequencies and sentiment of topics mentioned in the preliminary interviews with supervising physicians (absolute numbers and percentage).

No.	Topic	Neg.	Pos.	Total	% Neg.	% Pos.	% of total
1	Quality of training	13	67	80	16.3%	83.8%	14.3%
2	Duty scheduling	56	11	67	83.6%	16.4%	12.0%
3	Vacation scheduling	45	2	47	95.7%	4.3%	8.4%
4	Culture/atmosphere	37	20	57	64.9%	35.1%	10.2%
5	Personnel capacity	25	3	28	89.3%	10.7%	5.0%
6	Resources/equipment	25	11	36	69.4%	30.6%	6.4%
7	Leadership	3	24	27	11.1%	88.9%	4.8%
8	Performance orientation	15	1	16	93.8%	6.3%	2.9%
9	Onboarding	18	24	42	42.9%	57.1%	7.5%
10	Working environment	5	9	14	35.7%	64.3%	2.5%
11	Cooperation	10	5	15	66.7%	33.3%	2.7%
12	Technical services	13	2	15	86.7%	13.3%	2.7%
13	Administration	17	4	21	81.0%	19.0%	3.8%
14	Family/work life balance	8	3	11	72.7%	27.3%	2.0%
15	Quality of supervision	9	5	14	64.3%	35.7%	2.5%
16	Salary	1	1	2	50.0%	50.0%	0.4%
17	Career perspectives	16	2	18	88.9%	11.1%	3.2%
18	Flexible work schedules	3	6	9	33.3%	66.7%	1.6%
19	Cooperation with nurses	8	21	29	27.6%	72.4%	5.2%
20	Working hours	10	2	12	83.3%	16.7%	2.1%
	Sum/mean	337	223	560	62.9%	37.1%	100%

### 3.2. Study 2: main interview study

#### 3.2.1. Design

Using the same interview guide as in Study 1, we conducted semi-structured interviews with physicians. They were asked to talk about strengths, weaknesses and suggestions for improvement concerning the clinic in general, work environment, training, management, leadership, and cooperation. At the end of the interviews, all interviewees were asked to complete three quantitative NPS ratings: 1. willingness to recommend the clinic as a workplace, 2. willingness to recommend the clinic’s training program, and 3. probability to stay in the clinic for the next 5 years. They also reported how long they already had worked for the clinic and how long they had held their actual position. Interviews were recorded and transferred into short statements which were rated according to their sentiment (positive, negative) and their affiliation to a topic derived from the preliminary study.

#### 3.2.2. Participants

In summary, n = 46 physicians participated in the interviews, n = 13 female and n = 33 male persons. On average, they worked for the hospital for 7.04 years and held their current position for 3.25 years.

#### 3.2.3. Method and procedure

Interviews included the same questions as in the preliminary study (see Supplementary Appendix 1.2; original version in Supplementary Material 2). Interviews lasted about 30 to 140 min (mean 60 min) and were documented in a written protocol. Protocols were checked by the interviewees, who gave their written consent that the protocols may be used in this research. Protocols were anonymized and rated using the codebook developed in the preliminary study (see Table 1 and Supplementary Material 4). To measure interrater reliability, randomly picked 10 interviews were categorized by two independent raters. All items could be categorized and interrater reliability was *r* = 0.82 (Cohent’s Kappa), which indicates a sufficient reliable categorization of the items derived from the interviews (Yawn and Wollan, 2005). NPS rated ranged from 1 (recommendation very unlikely) to 10 (recommendation very likely). Recommendation scores were grouped according to the NPS scheme into three groups: Promoting (9–10), neutral (7–8), and skeptical (1–6).

#### 3.2.4. Results

##### 3.2.4.1. Structure and relation of topics

In total, 1,239 positive or negative statements were counted resulting in a frequency table which lists frequencies per topic (see Table 3). Most of the statements were negative (67.8%) and mostly addressed the quality of professional training (12.6%), duty (10.3%), and vacation (8.6%) scheduling and aspects of culture and atmosphere in the clinic (8.8%).

**TABLE 3:** Frequencies and sentiment of topics mentioned in the main interviews with residents and non-supervising physicians (absolute numbers and percentage).

No.	Topic	Neg.	Pos.	Total	% Neg.	% Pos.	% of total
1	Quality of training	30	126	156	19.2%	80.8%	12.6%
2	Duty scheduling	111	17	128	86.7%	13.3%	10.3%
3	Vacation scheduling	97	9	106	91.5%	8.5%	8.6%
4	Culture/atmosphere	71	38	109	65.1%	34.9%	8.8%
5	Personnel capacity	69	4	73	94.5%	5.5%	5.9%
6	Resources/equipment	40	31	71	56.3%	43.7%	5.7%
7	Leadership	9	49	58	15.5%	84.5%	4.7%
8	Performance orientation	41	1	42	97.6%	2.4%	3.4%
9	Onboarding	37	42	79	46.8%	53.2%	6.4%
10	Working environment	8	11	19	42.1%	57.9%	1.5%
11	Cooperation	32	7	39	82.1%	17.9%	3.1%
12	Technical services	18	15	33	54.5%	45.5%	2.7%
13	Administration	53	6	59	89.8%	10.2%	4.8%
14	Family/work life balance	16	4	20	80.0%	20.0%	1.6%
15	Quality of supervision	22	10	32	68.8%	31.3%	2.6%
16	Salary	30	1	31	96.8%	3.2%	2.5%
17	Career perspectives	43	2	45	95.6%	4.4%	3.6%
18	Flexible work schedules	7	10	17	41.2%	58.8%	1.4%
19	Cooperation with nurses	22	37	59	37.3%	62.7%	4.8%
20	Working hours	59	4	63	93.7%	6.3%	5.1%
	Sum/mean	815	424	1,239	67.8%	32.2%	

Topics in general hardly correlated with each other (see Supplementary Appendix 2). Exceptions were highly significant positive correlations (*p* < 0.001) between the statements concerning duty scheduling and work environment in general (*r* = 0.47), comments about quality of supervision and personnel capacity (*r* = 0.40), as well as salary and working hours (*r* = 0.40). Highly significant positive correlations (*p* < 0.001) were found between statements about working hours with cooperation in general (*r* = 0.49) and with salary (*r* = 0.40). However, there was a negative correlation of onboarding with career perspectives (*r* = −0.38).

In total, 232 different ideas and suggestions were generated by the interviewees which amounted to 166 statements when duplicates were removed. A majority of 96 suggestions were only mentioned once, 70 by more than one person, and a “top list” of 22 improvement ideas were proposed by five or more persons or more (see Supplementary Appendix 3). This top list was used to develop improvement measures in university hospitals participating in the multi-center study. The most popular suggestions with more than ten mentions each were: (1) Optimize employee appraisals, (2) making career prospects more transparent, (3) longer assignments (do not plug gaps and help out), (4) creating niches/specializations (e.g., outpatient clinic), (5) financial support for further training, and (6) continue rotation and introduce target agreement discussions.

##### 3.2.4.2. Net promoter score

Quartile groups were calculated concerning the number of positive and negative interview items (from 1 to 4, group 1 representing the lowest amount of positive and highest number of negative items). These group variables were used as independent variables, NPS as dependent variables in multiple analyses of variance (MANOVA). Table 4 summarizes variables used in the MANOVA. As expected, the grouped frequency of positive interviews items had a significant positive impact on all NPS ratings [work *F*(3,42) = 6,5, *p* < 0.01; training *F*(3,40) = 3.1, *p* < 0.05; intention to stay *F*(3,41) = 3.8, *p* < 0.05]. Negative interview items showed only significant main effect on retention [*F*(3,38) = 3.6, *p* < 0.05] and a significant interaction for work NPS: The more positive and less negative items were rated the higher was work satisfaction [*F*(1,7) = 3.3, *p* < 0.05].

**TABLE 4:** Mean frequency of cluster items as a function of promotors, neutrals, and skeptics according to net promotor score (NPS).

Net promotor score (NPS)	NPS group	Positive cluster items	Negative cluster items	Total cluster items
**Work**				
	Promotor	9.5	14.3	32.4
	Neutral	10.2	20.0	37.5
	Skeptic	7.9	17.7	33.6
**Education**				
	Promotor	8.9	17.3	34.6
	Neutral	9.9	19.4	35.9
	Skeptic	10.0	13.5	31.5
**Intention to stay**				
	Promotor	10.9	16.0	34.7
	Neutral	10.0	17.7	35.9
	Skeptic	8.0	18.2	33.9

To figure out which topics had a positive or negative influence on the NPS concerning work, training, and intention to stay we applied three canonical discriminant analyses. Frequencies of positive and negative statements were used to predict the three NPS groups “skeptical” (scores 1–6), “neutral” (7–8), and “promoting” (9–10) concerning the ratings about work, training, and intention to stay. Analyses about work and intention to stay classified 100% of the cases correctly, the analysis in view of training 97%. In all cases the discriminant factors explained 100% of the variance. We used Wilks’ lambda as test statistics (Klecka et al., 1980; AlKubaisi et al., 2019) to select those items that substantially contribute to the prediction of the NPS group (skeptical, neutral, promoting) concerning work, training, and intention to stay (*p* < 0.10; see Supplementary Appendix 4).

Absolute differences of the function coefficients between NPS groups indicate that aspects like workload, predictability of duty schedules, career perspectives, technical and administrative support, appreciation from and cooperation with colleagues and superiors as well as payment play an important role with either a clearly negative (poor career perspectives, low salary) or positive (excellent career outlook and sufficient income) connotation. In addition to these aspects, function coefficients for work-life balance, staff capacity, performance orientation, cooperation and career perspectives differ largely regardless of work training or intention to stay. All aspects with high frequency of positive mentions play a supportive role for the intention to stay. In contrast, the factor “salary” shows only a negative impact on the intention to stay, if the frequency of negative mentions is high. Based on the results of the discriminant analyses and on topics suggesting improvements we reduced the interview topic list to 14 top issues and ordered them in a polarity scale describing dissatisfiers on the one side and satisfiers on the other (see Table 5).

**TABLE 5:** Item statistics for polarity satisfaction scale.

	Mean of Likert scale (1–10)												
**Positive Item**	**1**	**2**	**3**	**4**	**5**	**6**	**7**	**8**	**9**	**10**	**Negative item**	**Std. dev.**	**Item-tot. corr.**
My work is appreciated and supported by my colleagues.		1.85									My work is hardly appreciated and unnecessarily criticized by my colleagues.	0.95	,220
My work is appreciated and supported by my superiors.		2.09									My work is hardly appreciated and unnecessarily criticized by my superiors.	1.06	,387
My superiors know my personal goals and take them into account as far as possible.			2.55								My personal goals are neither perceived nor taken into account by my superiors.	1.40	,559
You can always rely on promises made by the clinic and superiors.			2.99								Promises cannot be trusted because they are not kept.	5.56	,117
I will be informed in time when and where I have to work. I can make plans for the long term.			3.06								I won’t be informed in time when and where I have to work. I can‘t make plans for the long term.	1.80	,399
I will be informed sufficiently and early enough about plans and decisions that affect my work.			3.04								Often, I am not informed sufficiently and early enough about plans and decisions that affect my work.	1.49	,448
Decisions that affect my work as well as the decision-making process are easy for me to understand.			2.68								I often can’t understand decisions and decision-making processes at my workplace.	1.42	,391
Within the given framework, I can decide for myself how I do my work.		2.28									I have no room for own decisions and feel that I am being thwarted by the specifications in my work.	1.20	,148
The work offers many challenges, but I never feel overwhelmed.			2.51								I feel overwhelmed by the demands of my work.	5.51	,053
In the clinic, I do meaningful work that benefits society.		1.54↑									I do pointless work that is of no use to anyone.	0.94	,304
In the clinic I find working conditions that are important to me and that I could not find anywhere else.											I might as well work in another hospital.	1.62	,321
…colleagues.			2.68								I don’t get enough money for the work I do.	1.68	,355
…superiors.		2.45									I see the clinic as a dead end where I cannot develop professionally.	1.40	,403
My superiors know my personal goals and take them into account as far as possible.			3.43↓								In my experience, the workload is unbearable, and it won’t get better in the future.	1.19	,353

↑ = best valued item, ↓ = worst valued items (segments are colored from positive = green to negative = red).

##### 3.2.4.3. Cohort effects

All participants were grouped in quartiles according to the length of their stay in their current position, group 1 with the shortest and group 4 with the longest stay. Work experience had a great influence on work satisfaction and retention as can be seen in Table 6. As predicted, the results of the MANOVA showed that employees evaluated their work better the longer they worked in the clinic [*F*(3,12) = 3.3, *p* < 0.05], whereas the specific position did not have a significant impact. Unexpectedly, the longer employees stayed with the clinic, the more interview items were generated [*F*(3,42) = 3.4, *p* < 0.05] with a lower amount of negative items [*F*(3,42) = 2.9, *p* < 0.05]. This effect is validated when calculating the ratio of positive and negative items: The ratio of negative items declined with length of stay [*F*(3,42) = 4.2, *p* < 0.05].

**TABLE 6:** Means ratings for of willingness to recommend (net promotor score, NPS) as a function of career stage (and standard deviation).

	Willingness to recommend		
**Career stage**	**NPS work**	**NPS training**	**NPS intention to stay**
Resident in training	7.86 (1.89)	7.39 (2.28)	5.33 (3.47)
Resident at/after end of training	6.27 (2.60)	7.23 (1.66)	4.14 (2.68)
Non-supervising physician	7.42 (1.75)	6.13 (2.33)	5.52 (2.69)
Supervising physician	8.35 (1.94)	7.24 (1.64)	5.47 (3.76)
Total	7.43 (2.12)	6.96 (2.13)	5.16 (3.16)

### 3.3. Study 3: mental maps from repertory grids

#### 3.3.1. Design

Job satisfaction is a highly individual construct changing largely over different career stages (Gordon, 2017). We, therefore, used the Repertory Grid technique in order to create “mental maps” visualizing personal mental constructs concerning work satisfaction (Winter, 2003; Hill et al., 2016). In addition, we checked for changes of NPS regarding different career stages. To this end, we used the polarity scale of job satisfiers and dissatisfiers derived from the main interview study (see Table 5) to predict NPS concerning work, training, and intention to stay.

#### 3.3.2. Participants

In order to generalize from our previous results, we included more university hospitals and employees in the main study. We recruited n = 112 physicians, n = 48 female, and n = 64 male persons, working in four university hospitals, who did not participate in one of the preceding studies. On average, they held their positions for 5.2 years and worked for their hospital for 6.9 years. The participants’ positions covered the complete range of career steps: n = 40 held the position of supervised residents in training stage, and n = 24 at the end of their vocational development or shortly after working as assistant physicians, n = 31 persons were non-supervising physicians and n = 17 were supervising physicians.

#### 3.3.3. Method and procedure

**FIGURE 2:**
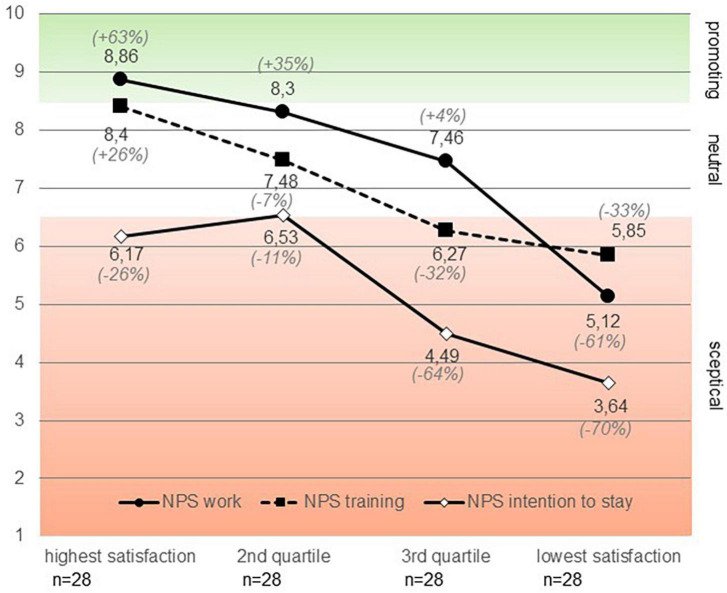
Rating of willingness to recommend work conditions, training, and intention to stay (and NPS in %) as a function of quartile groups of mean of the polarity satisfaction scale.

All participating physicians were invited to the study by their chief physicians and received a comprehensive document about the study procedure. Subjects then expressed their consent via email. No names or other identifiable features were recorded. Participants filled in a brief NPS survey concerning their work satisfaction, satisfaction with training and personal development, their intention to stay in the clinic for the next 5 years as well as the polarity satisfaction scale (cf. Figure 2). Repertory Grids were performed as an anonymized, computer-based survey in the procedure described above (see section “2.2. Material and procedure in general”). The entire computer-based survey took 20 to 30 min.

#### 3.3.4. Results

##### 3.3.4.1. Net promoter score (NPS)

We calculated NPS groups concerning work conditions, training, and personal development as well as intention to stay in the clinic for at least the next 5 years. The NPS concerning work conditions [*F*(3,106) = 3.75; *p* < 0.05] and training (*F*(3,106) = 3.23]; *p* < 0.05] differed significantly across hospitals. Working conditions in anesthesiologic clinics in general differ to a large extent in terms of size, scope, cooperation with other disciplines and staff structure (Hinkelmann et al., 2018). Thus, the following analysis was calculated with the identification number of the hospital as a covariate to control for this source of variance. Using means of the job satisfaction scale we calculated four quartile groups (very satisfied, satisfied, dissatisfied, and very dissatisfied). We took the four groups as independent variables and NPS ratings as dependent variables. NPS scores for work [*F*(3,106) = 24.56; *p* < 0.001], training [*F*(3,106) = 8.78; *p* < 0.001], and intention to stay [*F*(3,106) = 4.85; *p* < 0.01] differed significantly as a function of job satisfaction group (see Figure 2). As shown in Figure 2, only ratings concerning work and training by subjects with high job satisfaction supported a promoting attitude. We also calculated the NPSs in percent, that is, the percentage of promotors (scoring 9 or 10 on the NPS scale) minus the percentage of skeptics (scoring 6 or below). The NPSs concerning work conditions were mostly positive with a sharp decline for the least satisfied group (from −61% to + 63%, average + 10%). The pattern was just opposite for the NPSs concerning training: Only the most satisfied quartile group showed a positive NPS (+ 26%, ranging from −33% to + 26%, average −12%).

##### 3.3.4.2. Satisfaction scale and NPS

The 14 items satisfaction scale had a satisfactory reliability of Cronbach’s alpha = 0.78. In other studies the same scale within the PhysicianPlus project (Hasebrook et al., 2016b; Hinkelmann et al., 2017; Hahnenkamp et al., 2018) as well as in another project (Hasebrook et al., 2022) the scale was found to be highly reliable between an Cronbach’s Alpha of 0.91 to 0.78 (Hahnenkamp et al., 2018). In this study, items show low intercorrelations (see Supplementary Appendix 5). In total, the mean ratings of the items indicate a positive attitude and vary between 1.5 and 3.4 (with 1 o 5 indicating a more positive and 6 to 10 a more negative evaluation) with “purposeful work” being the best and “demanding working hours” the worst evaluation (see Table 5).

We checked to what extent the satisfaction scale can predict NPS ratings. To this end, we calculated a discriminant analysis summarizing all three NPS ratings summing up how many times a person promoted work, training, or intention to stay from 0 (no promotion) to 3 (promoting all three aspects). The results show that regarding personal goals, providing sufficient information, making comprehensible decisions by superiors, opening career perspectives, and providing a good working environment were the most important factors differentiating between promotors and non-promoters (full results are listed in Supplementary Appendix 6).

##### 3.3.4.3. Repertory grids

**FIGURE 3:**
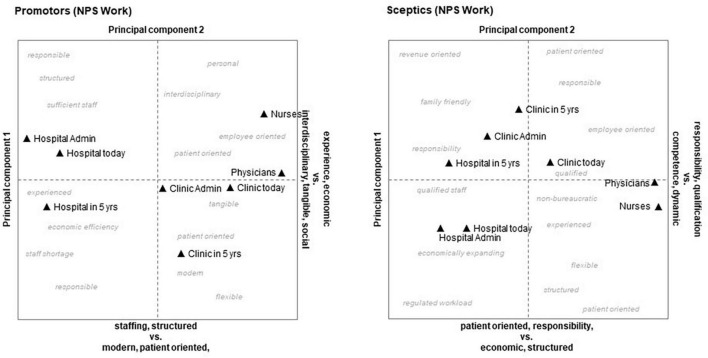
Kelly grids of promotors **(left)** and skeptics **(right)** regarding “recommending work” net promotor score (NPS).

**FIGURE 4:**
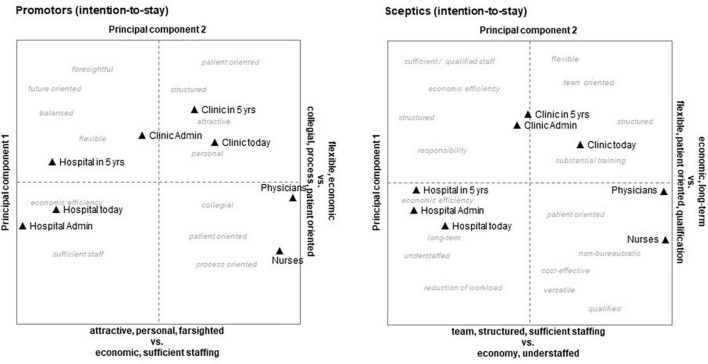
Kelly grids of promotors **(left)** and skeptics **(right)** regarding retention (intention-to-stay).

Using the Kelly Grids method, we got representations of “mental landscapes” showing the mental distance between elements (with reference to a specific definition), and whether they were rated more positively or negatively. Figures 3, 4 contain orthogonal coordinate systems with the extracted factors of the first two components (full details of the factor analysis can be found in Supplementary Appendix 7). An interesting difference between promotors and skeptics concerning work was that promotors created a “we and others” position with physicians, clinic, and clinic administration close together in contrast to hospital administration and hospital in general. Skeptical persons, however, tended to distinguish between medical staff (physicians and nurses) and the rest. Persons with high intention to stay had a more positive perception of the university hospital’s future (element “Hospital in 5 years”) than employees with low intention to stay. They connected the hospital’s future with positive features like “foresightful,” “future oriented,” and “balanced” as compared to skeptics, who chose characteristics like “sufficient staff,” “economic efficiency,” and “structured.”

## 4. Discussion

In this study we asked, how individual statements and mental maps about job (dis)satisfaction derived from physicians in academic medicine can be structured, and how these individual perspectives influence intention to stay and turnover intention. We were also interested to learn, whether summing up individual perspectives support or–even partly–contradict the model-based research findings based on standardized tools (Hayes et al., 2020).

### 4.1. Summary and research questions

We found that negative interviews statements clearly outweigh positive ones and mostly address the quality of professional training, duty and vacation scheduling, and aspects of culture and work atmosphere in the clinic. Correlation patterns indicate that the evaluation of work environment is mainly influenced by the quality of duty scheduling and other administrative issues. Workload depends on the quality of cooperation and is seen directly according to the salary paid for it. Career perspectives are linked to the quality of the onboarding process and quality of supervision depends on a sufficient staff capacity. This is very much in line with other research findings [overview in Coomber and Barriball (2007), Aziri (2011), Domagała et al. (2018)].

Individual statements, negative (weaknesses) and positive (strengths) are connected to the individual valuation of work and training quality and the intention to stay. The frequency of positive items found in the interviews had a significant positive impact on all NPS ratings concerning work, training, and intention to stay. In contrast, negative items showed only significant main effect on retention and a significant interaction. The more positive and less negative items were counted, the higher was work satisfaction. In summary, all aspects with high frequency of positive mentions play supportive role for the intention to stay. However, the factor “salary” shows only a negative impact on the intention to stay if the frequency of negative mentions is high. This finding supports the function of “salary” as dissatisfier or hygiene factor (Herzberg, 1965; Kao et al., 2018).

Individual valuations of working conditions are clearly affected by cohort effects, such as work experience and career stage. Work experience had a great influence on work satisfaction and retention, whereas the specific position did not have a significant impact: The longer employees stayed with the clinic, the lower the amount and the ratio of negative items. Our study suggests, NPS ratings of physicians employed in academic medicine are low in comparison to other studies: A low positive NPS can be expected from 0% up to + 15% (West et al., 2009; Sieja et al., 2019). However, in this study the NPS concerning intention to stay was always rated negative regardless how high or low job satisfaction was (NPS from −26 to −70% with an average intention to stay of −42%).

We summarized positive and negative interviews items in a polarity satisfaction scale. It is noteworthy that all significant intercorrelations of the polarity satisfaction scale between the items of the satisfaction scale were positive, suggesting that all items are positively connected to a general concept of job satisfaction. The item with the highest intercorrelations with almost all other items was “regard of personal goals,” whereas the item “reliability of promises kept” was not significantly interconnected to other items. This may indicate that in employees’ opinions a clinic regarding personal goals causes a positive attitude toward work whereas broken promises are singular events playing a negative role as “hygiene factor” (Herzberg, 1965; Domagała et al., 2018; Jackson et al., 2018; Johna, 2018).

The “mental maps” generated from the Repertory Grid procedure revealed that persons with high intention to stay had a more positive perception of the university hospital’s future than employees with low intention to stay. They connected the hospital’s future with positive features (e.g., “future oriented”) as compared to skeptics, who chose characteristics with negative connotations (e.g., “economic efficiency”). Comparing “job promotors” to “job skeptics” by repertory grids indicates that an important difference between these two groups may lie in a “hope for a better future” and high affinity for the employer on the one hand and hopelessness as well as a notion of extraneousness at work on the other. Highly trained and qualified physicians, like the subjects in this study, are more attached to their profession and their discipline than to the actual clinic or hospital, for which they work (Choi et al., 2011). Moreover, the mental maps generated by Repertory Grids procedures in our study show that the very same factors may be seen as negative by some individuals and positive by others (Younge and Marx, 2016; Mathimaran and Kumar, 2017).

### 4.2. Limitations and future research

Open interviews can lead to new insights because the range of questions and answers is not limited. Standardized instruments measuring job satisfaction and job retention limit the factors and interactions, which can be identified (e.g., Singh, 2019; Rombaut and Guerry, 2020). Several standardized tools are available from scientifically validated tests (e.g., Van Saane et al., 2003) and commercial testing packages (e.g., Riechmann and Stahl, 2013). All standardized tools necessarily operate with a standard set of questions or items restricting the range of potential answers and justifying the use of our interview format for explorative purposes. In the interviews reported here, the participants did not bring up many new topics and interrelations, and the results mostly support existing research findings. However, self-reports are an important source for a more differentiated understanding of quantitative research results (Choi et al., 2011; Alrawashdeh et al., 2021). Unfortunately, conducting and evaluating qualitative interview data is a highly resource intensive task and cannot be implemented as a routine evaluation process. As a result, the relatively low number of subjects, especially in Study 1 (*n* = 20) and Study 2 (*n* = 46), is a limitation of our research. We investigated physicians in university hospitals. On the one hand, this limits the scope of our research because results cannot easily be transferred to other clinical settings or healthcare workers. On the other hand, it adds to a better understanding of the specific working conditions and needs in academic medicine (Hahnenkamp et al., 2018).

In order to combine standardized quantitative and open qualitative study methods we applied Repertory Grids as a quantitative method to describe qualitative data. The results derived from this computer-led survey technique very much depend on the usability and visual front-end of the software applied. In summary, employee surveys need careful planning in view of how efficient and standardized the inquiry may be and how open and explorative it has to be.

Our studies are not sufficient to measure the impacts of various factors influencing job satisfaction and intentions to stay as well as the impacts of interrelations due to the methods used. We were only able to identify the factors generally impacting the matter. For distinguishing the different impacts of factor, e.g., whether high workload has a bigger impact on job dissatisfaction than unpredictable staff planning, further research with alternative methodical approaches is necessary. It should also be noted that the study has been conducted prior to and in preparation of an intervention study. This setting may have biased the interviews, especially the suggestions for improvements.

### 4.3. Practical implications

The most popular suggestions for improvements from the physicians in our studies were optimization of employee appraisals, more transparent career prospects, longer assignments for a certain position in the clinic, niches for specializations, financial support for further training, and planned discussions about target agreements. This suggests that the focus of improvements does not lay in individual support but in structural adaptations. During the PhysicianPlus project four aspects to foster intention to stay and to lessen turnover intention were developed and afterward implemented: (1). Employee appraisal, (2). training in fellowship programs, (3). computer-assisted duty and vacation scheduling, and (4). corporate benefits.

#### 4.3.1. Employee appraisal to further career prospects and transparency

Better and more frequent feedback and employee appraisal was the most suggested improvements. Referring to samples of employee appraisal interviews in professional service firms—consultants, lawyers, and financial services—a PhysicianPlus interview guide has been implemented based on the polarity satisfaction scale. Both the interview guide and the satisfaction scale are in use in several university hospitals and have already made their way into some smaller hospitals, as well (Spiegelberg, 2022). Users report that the guide helps them to lead structured and focused interviews providing employees with a clear understanding of their strength, needs for further development, and career prospects, such as participating in sought-after training programs.

#### 4.3.2. Specialized training courses as a fellowship program

The positive aspect with most mentions was training and career development. Therefore, we implemented highly specialized training curricula in the style of fellowship programs carried out in hospitals outside Germany: Experts from hospitals in the Netherlands, United Kingdom, United States, and New Zealand reported about their programs. In the PhysicianPlus project, three fellowship programs started with up to twelve attendees each: Neuro-anesthesia, cardio-anesthesia, and special child-anesthesia. Participants within these training programs reported that they were willing to stay in the hospital for another 1 or 2 years when their participation is guaranteed.

#### 4.3.3. Improved duty and vacation scheduling

The aspect with the most negative mentions was duty and vacation scheduling. Further investigation showed that two aspects cause dissatisfaction: Unreliable time planning (e.g., changes on short notice), and mental under- or overload when individual skills and competences did not match job demands. Human planers are not able to consider all organizational and individual demands, thus, coming up with poor plans. Drawing from highly sophisticated workforce planning systems in airport and harbor logistics, we created algorithms for vacation and duty scheduling. The vacation planning supports fair and more transparent vacation planning considering aspects such as private care (e.g., young children or elderly parents) or sacrificing vacation plans to support the clinic in prior seasons. We also developed a competence-based workforce planning model for physicians in large hospitals. As a proof-of-concept we implemented the model as a SQL database (which can be accessed from Supplementary Material 5). The PhysicianPlus planning model has been adopted by professional workforce planning software.

#### 4.3.4. Corporate benefits

The interviewees generated a great variety of good ideas, which sometimes were tailored to the specific situation of the hospital, for which they were working. Therefore, we encouraged minor but meaningful changes, such as providing drinking water and healthy snacks in a central operation theater, a comfortable lounge for physicians for relaxation, informal chats, and seatwork. Several hospitals adopted a corporate benefit program from professional service firms providing employees with valuable goods and services at reduced prices (Hahnenkamp and Hasebrook, 2022). These hospitals reported that their employees valued the benefit program as a special sign of appreciation for their work.

## 5. Conclusion

Just eliminating factors which elicit job dissatisfaction does not automatically lead to higher satisfaction. Whereas, high workload and unpredictable staff planning plus duty rosters lead to dissatisfaction, recognition by superiors as well as regular and systematic appraisal interviews are the most important factors driving job satisfaction. The interview data and discriminant analyses show a high stability of reasons to leave—mostly referring to unfavorable management, staff planning, workload and low income. It also shows that reasons to stay are highly individualized, such as regarding personal goals. Decisions rely on a balance between perceived efforts to stay and gains from the job, e.g., work-family culture and further education offered by the university hospital. This balance is hard to maintain when personal growth by means of medical trainings decreases during the career, but workload increases. The position as a chief physician seems to offer the best balance and highest retention with less strenuous work (e.g., no swing shifts), better income, and more authority.

As many studies present a close connection between job satisfaction and retention, the general recommendation is to increase satisfaction to increase the retention of medical staff (Nantsupawat et al., 2017; Jackson et al., 2018). Systematic literature research demonstrates that workload, stress and leadership affect dissatisfaction and turnover, but the results for factors associated with job satisfaction are not consistent (Coomber and Barriball, 2007; Johna, 2018). Likewise, there is no connection between job satisfaction and retention supported by our data: Minimizing dissatisfaction does not automatically lead to more satisfaction and more satisfaction does not necessarily lead to higher retention. This appears especially true for physicians in university hospitals who are a highly qualified and mobile workforce, who show higher identification with their job and medical discipline than with their employer. Consequently, improvements suggested by the physicians in our study focused on personal growth and individual work-life-balance and not on eliminating dissatisfaction factors like poor career prospects. As a result, university hospitals reacted offering regular and systematic appraisal interviews and individual mentoring for all physicians as well as a wide range of other measures ranging from bonus programs to fellowships for specialized trainings (Nasir and Mahmood, 2018). Mentoring provided by experienced supervising physicians leads to highest satisfaction and retention scores. These mentors also help to select the right measures to meet the needs of both the individual physicians and the hospital in general.

As demonstrated in other research, study results of this research effort indicate that team coherence (Kim and Yi, 2019; Zaheer et al., 2019) and hope for improvement concerning work environment (Tummers et al., 2013; Rombaut and Guerry, 2020) are the main aspects to retain highly skilled staff. Active retention management is needed but currently underrated and not carried out systematically (Singh, 2019). As intention to leave is recognized easily, regular, brief employee surveys help to identify human resource risks in advance. Intentions to stay are highly individual and cannot be answered with a small set of measures. A systematic set of retention measures is needed (Gerson, 2002; Verlander and Evans, 2007) which helps hospital management developing individually tailored activities in order to satisfy individual demands of high skill workers.

## Data availability statement

The datasets presented in this study can be found in online repositories. The names of the repository/repositories and accession number(s) can be found below: https://www.researchgate.net/publication/365616201_Data_PhysicianPlus_Main_Interview_Study.

## Ethics statement

Ethical review and approval was not required for the study on human participants in accordance with the local legislation and institutional requirements. The patients/participants provided their written informed consent to participate in this study.

## Author contributions

All authors listed have made a substantial, direct, and intellectual contribution to the work, and approved it for publication.

## Supplementary material

Supplementary Table 1 Coding Book Interview Studies 1 + 2.

Supplementary Data Sheet 1 Interview Guide Study 2 + 3 (German Original).

Supplementary Data Sheet 2 Interview Guide Study 3 (German Original).

Supplementary Data Sheet 3 Interview Result Protocol (German Original plus English Translation).

Supplementary Data Sheet 4 Access Database with Skills Matching System.

Supplementary Data Sheet 5 Supplementary Appendices.
