# Supplementary material for: Individual perspectives and mental maps of working conditions and intention to stay of physicians in academic medicine
Source: Front Psychol. 2023 May 12;14:1106501. doi: 10.3389/fpsyg.2023.1106501 (PMC10213555; doi:10.3389/fpsyg.2023.1106501)
Supplement: Supplementary Data Sheet 1 — Interview Guide Study 2 + 3 (German Original). [file Data_Sheet_1.PDF]

Facharzt+

Einladung zum Interview

Liebe Fachärztinnen und Fachärzte,

die erste Phase unseres Vorhabens *FacharztPlus* läuft in vollen Zügen. Wie bereits angekündigt möchten wir mit dem gemeinsamen Projekt erreichen, dass sich Ihre Arbeit noch stärker an Ihren individuellen Bedürfnissen, Kompetenzen und Zielen ausrichtet.

Zentral für den Erfolg dieses Vorhabens ist daher vor allem Ihre Meinung und aktive Mitarbeit. Die Basis dafür möchten wir über ein erstes Gespräch schaffen, bei dem wir Ihre Meinungen, Anregungen und Wünsche zum Projekt *FacharztPlus* einholen und Sie persönlich kennenlernen möchten. Themen sind u.a. die Besonderheiten des UKM und Ihrer Klinik hinsichtlich Organisation und Führung.

Wir würden uns sehr freuen, wenn Sie uns dazu im Verlauf der nächsten Wochen für ein jeweils 30-45 minütiges Einzelgespräch zur Verfügung stehen. Für eine bessere Koordination der Gespräche, haben wir eine Terminübersicht erstellt und möchten Sie bitten, sich unter Angabe Ihres Namens Ihren Wunschtermin einzutragen. Alle Eingaben erfolgen anonymisiert und sind für Ihre Kolleginnen und Kollegen nicht sichtbar. Eine separate Terminbestätigung senden wir Ihnen anschließend per Email zu.

An dieser Stelle möchten wir nochmals betonen, dass die Ergebnisse der Gespräche streng vertraulich behandelt und ausschließlich für das Projekt *FacharztPlus* verwendet werden. Die Auswertung wird nur summarisch erfolgen, womit keine Rückschlüsse auf Ihre Person gezogen werden können. Die im Nachgang zum Interview angefertigten Protokolle erhalten Sie zugesandt und können Anmerkungen dazu machen. Die Ergebnisse werden erst nach Ihrer schriftlichen Freigabe genutzt.

Wir freuen uns auf Ihr Mitwirken und die Gespräche Ihnen!

Mit freundlichen Grüßen

GEFÖRDERT VOM
Bundesministerium für Bildung und Forschung
UKM Universitätsklinikum Münster
zeb/

Translation of the German original

Dear physicians,

the first phase of our *Project PhysicianPlus* is running to the fullest. As already announced, we want to achieve with the joint project that your work is even more oriented to your individual needs, competencies and goals.

Central to the success of this project is therefore above all your opinion and active cooperation. We would like to create the basis for this through an initial conversation, in which we would like to obtain your opinions, suggestions and wishes for the *PhysicianPlus* project and get to know you personally. Topics include the special features of the UKM (University Hospital Muenster) and your clinic in terms of organization and leadership.

We would be very pleased if you would be available to us in the course of the next few weeks for a 30-45 minute one-on-one conversation. For a better coordination of the discussions, we have created an overview of the dates and would like to ask you to enter your desired date stating your name. All entries are anonymous and are not visible to your colleagues. We will then send you a separate appointment confirmation by email.

At this point, we would like to emphasize once again that the results of the discussions are treated as strictly confidential and used exclusively for the *FacharztPlus* project. The evaluation will only be carried out summarily, which means that no conclusions can be drawn about your person. The minutes made after the interview will be sent to you and can make comments on them. The results will only be used after your written approval.

We look forward to your cooperation and the discussions with you!

Sincerely,

Verbundprojekt FacharztPlus
Initialgespräche mit Fachärztinnen und Fachärzten

- Gesprächsleitfaden -

Gesprächspartner

Funktion: ______________________

UKM seit: ______________________

Facharzt seit: ______________________

Interviewer: ______________________

Datum/Zeit: ______________________

GEFÖRDERT VOM
Bundesministerium
für Bildung
und Forschung

Persönliche Vorstellung

- Was sind Ihre Aufgaben innerhalb der Klinik?

Besonderheiten UKM/Klinik

- Was erleben Sie als besondere Stärke Ihrer Klinik?

- Wo sehen Sie Schwachstellen?

- Haben Sie Vorschläge, wie man den Schwächen entgegenwirken könnte?

- Würden Sie einem Freund oder Kollegen von einer anderen Klinik empfehlen in Ihrer Klinik als Facharzt zu arbeiten?
  (0 = sehr unwahrscheinlich; 10 = äußerst wahrscheinlich)

0	1	2	3	4	5	6	7	8	9	10
☐	☐	☐	☐	☐	☐	☐	☐	☐	☐	☐

- Würden Sie einem Freund oder Arzt von einer anderen Klinik empfehlen in Ihrer Klinik die Weiterbildung zum Facharzt zu absolvieren?
  (0 = sehr unwahrscheinlich; 10 = äußerst wahrscheinlich)

0	1	2	3	4	5	6	7	8	9	10
☐	☐	☐	☐	☐	☐	☐	☐	☐	☐	☐

- Kennen Sie Gründe, warum Fachärzte die Klinik verlassen haben?

- Was müsste getan werden, damit Fachärzte nach Abschluss ihrer Weiterbildung für einen längeren Zeitraum (2-5 Jahre) weiter an der Klinik arbeiten?

- Wie könnte man das erreichen?

- Wie erleben Sie die Zusammenarbeit mit den unterschiedlichen Angestelltengruppen (Pflegende, Verwaltung, technischer Dienst)?
- Wie beurteilen Sie die Zusammenarbeit zwischen Ärzten in allen Hierarchiestufen?

Organisation und Führungskultur

- Wie beurteilen Sie die Prozesse zur Personalplanung an Ihrer Klinik (Planung der Dienste, Urlaub, Zusatzqualifikationen)?

- Was könnte man aus Ihrer Sicht besser machen?

- Gibt es an Ihrer Klinik ausreichend Ressourcen, um Ihre Aufgaben erfolgreich zu bewältigen (Personal, Material, Geräte, finanzielle Mittel)?

____

____

- Welche regelmäßigen Formen der Personalführung und Personalunterstützung erleben Sie (z. B. Rückmelde-, Beurteilungs- oder Entwicklungsgespräch mit dem / der Vorgesetzten)?
- Was würden Sie gerne verbessern oder ergänzen?

____

____

____

Personalentwicklung

- Wie haben Sie Ihre eigene Einarbeitung an der Klinik erlebt? Was würden Sie ggf. verbessern?

____

____

- Wie gut ist aus Ihrer Sicht die Einarbeitung nicht deutschsprachiger Kolleginnen und Kollegen? Was sollte verbessert werden?

____

____

GEFÖRDERT VOM
Bundesministerium für Bildung und Forschung
UKM Universitätsklinikum Münster
zeb/

Perspektive der Fachärzte im UKM

- Finden Sie die Ziele des Projekt „FacharztPlus“ gut?
- Welche Ziele sollten ggf. ergänzt oder angepasst werden?
- Wie möchten Sie über Projektergebnisse informiert werden?

____

____

- Glauben Sie, dass Sie in fünf Jahren noch für diese Klinik arbeiten werden?
  (0 = sehr unwahrscheinlich; 10 = äußerst wahrscheinlich)

0	1	2	3	4	5	6	7	8	9	10
☐	☐	☐	☐	☐	☐	☐	☐	☐	☐	☐

- Haben Sie zum Abschluss unseres Gesprächs noch Kommentare oder Hinweise zum Projekt FacharztPlus?

____

____

____

Translation of the German original

Joint project PhysicianPlus
Initial discussions with specialists

- Conversation Guide -

Interlocutors

Function:
UKM since:
Specialist since:

Interviewer:

Date/Time:

Personal introduction

- What are your tasks within the clinic?

Special features UKM/Clinic

- What do you experience as a special strength of your clinic?
- Where do you see vulnerabilities?
- Do you have any suggestions on how to counteract the weaknesses?

- Would you recommend a friend or colleague from another clinic to work as a specialist in your clinic?
  (0 = very unlikely; 10 = extremely likely)

0	1	2	3	4	5	6	7	8	9	10
☐	☐	☐	☐	☐	☐	☐	☐	☐	☐	☐

- Would you recommend a friend or doctor from another clinic to complete further training as a specialist in your clinic?
  (0 = very unlikely; 10 = extremely likely)

0	1	2	3	4	5	6	7	8	9	10
☐	☐	☐	☐	☐	☐	☐	☐	☐	☐	☐

- Do you know any reasons why specialists have left the clinic?

- What would have to be done so that specialists continue to work at the clinic for a longer period of time (2-5 years) after completing their further training?

- How could this be achieved?

- How do you experience the cooperation with the different employee groups (nurses, administration, technical service)?
- How do you assess the cooperation between doctors at all hierarchical levels?

Organization and leadership culture

- How do you assess the personnel planning processes at your clinic (planning of services, holidays, additional qualifications)?

- What could be done better from your point of view?

- Are there enough resources at your clinic to successfully cope with your tasks (personnel, materials, equipment, financial resources)?

- What regular forms of personnel management and support do you experience (e.B feedback, appraisal or development interview with the supervisor)?
- What would you like to improve or supplement?

Staff development

- How did you experience your own induction at the clinic? What would you possibly improve?

- From your point of view, how good is the induction of non-German-speaking colleagues? What should be improved?

Perspective of specialists in the clinic

- Do you like the goals of the "FacharztPlus" project?
- Which goals should be supplemented or adapted if necessary?
- How would you like to be informed about project results?

- Do you think that you will still work for this clinic, 5 years ago?
  (0 = very unlikely; 10 = extremely likely)

0	1	2	3	4	5	6	7	8	9	10
☐	☐	☐	☐	☐	☐	☐	☐	☐	☐	☐

- At the end of our conversation, do you have any comments or hints about the Project PhysicianPlus?

GEFÖRDERT VOM
Bundesministerium für Bildung und Forschung
