# Supplementary material for: Individual perspectives and mental maps of working conditions and intention to stay of physicians in academic medicine
Source: Front Psychol. 2023 May 12;14:1106501. doi: 10.3389/fpsyg.2023.1106501 (PMC10213555; doi:10.3389/fpsyg.2023.1106501)
Supplement: Supplementary Data Sheet 2 — Interview Guide Study 3 (German Original). [file Data_Sheet_2.PDF]

Einladung zum Gespräch

Sehr geehrte Dame, sehr geehrter Herr,

wir schreiben Ihnen aus dem Büro des Verbundprojekts FacharztPlus. Wie bereits angekündigt, würden wir uns freuen, wenn Sie uns im Zuge des Projekts in nächsten Wochen für ein ca. 60-minütiges Einzelgespräch zur Verfügung stehen könnten.

Dabei möchten wir Sie gerne zu Ihren Erfahrungen und Wünschen hinsichtlich Ihres Arbeitsalltags befragen. Das Gespräch orientiert sich dabei zum einen an einem Leitfaden, zum anderen werden wir einen Teil des Interviews mit Softwareunterstützung durchführen. Mit Ihrer Teilnahme an diesem Gespräch, schaffen Sie eine wichtige Grundlage für die weitere Projektarbeit und tragen aktiv zum Erfolg des Projekts bei.

Für die Koordination der ersten Gespräche haben wir eine Terminübersicht erstellt und möchten Sie bitten, sich unter Angabe Ihres Namens Ihren Wunschtermin einzutragen. Alle Eingaben erfolgen anonymisiert und sind für Ihre Kolleginnen und Kollegen nicht sichtbar. Eine Terminbestätigung senden wir Ihnen anschließend per E-Mail zu. Sollte Ihnen keiner der vorgeschlagenen Termine zusagen, teilen Sie uns bitte einen möglichen Alternativtermin mit.

An dieser Stelle möchten wir nochmals betonen, dass die Ergebnisse der Einzelgespräche streng vertraulich behandelt und ausschließlich im Rahmen des Projekts FacharztPlus verwendet werden. Die Auswertung wird nur summarisch erfolgen, womit keine Rückschlüsse auf Ihre Person gezogen werden können.

Bei Fragen zum Projekt oder zu den Interviews kommen Sie gerne auf uns zu. Eine Übersicht über Projektziele und -vorgehen finden Sie anbei.

Wir freuen uns auf Ihr Mitwirken und das Gespräch mit Ihnen!

Mit freundlichen Grüßen

GEFÖRDERT VOM
Bundesministerium für Bildung und Forschung

Verbundprojekt FacharztPlus
- Gesprächsleitfaden -

Gesprächspartner

Funktion/Position: ______________________

An der Klinik seit: ______________________

In derzeitiger Position seit: ______________________

Interviewer: ______________________

Datum/Zeit: ______________________

Besonderheiten der Klinik

- Was erleben Sie als besondere Stärke Ihrer Klinik?

____

____

____

____

- Wo sehen Sie Schwachstellen innerhalb Ihrer Klinik?

____

____

____

____

- Würden Sie einem Freund oder Kollegen empfehlen an Ihrer Klinik zu arbeiten? (0 = sehr unwahrscheinlich; 10 = äußerst wahrscheinlich)

0	1	2	3	4	5	6	7	8	9	10
☐	☐	☐	☐	☐	☐	☐	☐	☐	☐	☐

- Würden Sie einem Freund oder Kollegen die Fort- und Weiterbildung an Ihrer Klinik empfehlen? (0 = sehr unwahrscheinlich; 10 = äußerst wahrscheinlich)

0	1	2	3	4	5	6	7	8	9	10
☐	☐	☐	☐	☐	☐	☐	☐	☐	☐	☐

- Wie begründen Sie Ihre Empfehlungen?

____

____

____

____

GEFÖRDERT VOM
Bundesministerium für Bildung und Forschung

Wie erleben Sie Ihren persönlichen Arbeitsalltag an der Klinik?
(1 = Aussage links, 10 = Aussage rechts oder ein Wert dazwischen)

**1.**	Für meine Arbeit bekomme ich ausreichend Wertschätzung und Unterstützung von…		Meine Arbeit wird wenig wertgeschätzt und unnötig kritisiert von…
1.1	…Kollegen.	1 2 3 4 5 6 7 8 9 10	…Kollegen.
1.2	…Vorgesetzten.	1 2 3 4 5 6 7 8 9 10	…Vorgesetzten.
**2.**	Meine Vorgesetzten kennen meine persönlichen Ziele und berücksichtigen diese, soweit möglich.	1 2 3 4 5 6 7 8 9 10	Meine persönlichen Ziele werden durch meine Vorgesetzen weder wahrgenommen noch berücksichtigt.
**3.**	Auf Zusagen, die die Klinik und Vorgesetzte gemacht haben, kann man sich stets verlassen.	1 2 3 4 5 6 7 8 9 10	Zusagen kann man nicht trauen, weil sie nicht eingehalten werden.
**4.**	Wann und wo ich arbeiten muss, kann ich langfristig planen.	1 2 3 4 5 6 7 8 9 10	Arbeitsorte und -zeiten ändern sind für mich nicht planbar.
**5.**	Über Planungen und Entscheidungen, die meine Arbeit betreffen, werde ich rechtzeitig und ausreichend informiert.	1 2 3 4 5 6 7 8 9 10	Über für mich wichtige Planungen und Entscheidungen werde ich oft nicht rechtzeitig und ausreichend informiert.
**6.**	Entscheidungen, die meine Arbeit betreffen, sowie der Entscheidungsprozess sind für mich gut nachvollziehbar.	1 2 3 4 5 6 7 8 9 10	Entscheidungen und Entscheidungsprozesse kann ich oft nicht nachvollziehen
**7.**	Im vorgegebenen Rahmen kann ich selbst bestimmen, wie ich meine Arbeit mache.	1 2 3 4 5 6 7 8 9 10	Ich habe keinen Entscheidungsspielraum und fühle mich in meiner Arbeit durch Vorgaben gegängelt.
**8.**	Die Arbeit bietet zwar viele Herausforderungen, ich fühle mich dabei aber nie überfordert.	1 2 3 4 5 6 7 8 9 10	Durch die Anforderungen meiner Arbeit fühle ich mich überfordert.
**9.**	In der Klinik und im UKM mache ich eine sinnvolle Arbeit, die der Gesellschaft nützt.	1 2 3 4 5 6 7 8 9 10	Ich verrichte sinnlose Arbeiten, die niemandem nützen.
**10.**	In der Klinik und im UKM finde ich Arbeitsbedingungen, die mir wichtig sind, und die ich nirgendwo sonst finden könnte.	1 2 3 4 5 6 7 8 9 10	Ich könnte genauso gut in einem anderen Krankenhaus arbeiten.
**11.**	Für meine Arbeit werde ich gerecht und angemessen bezahlt.	1 2 3 4 5 6 7 8 9 10	Für die Arbeit, die ich leiste, werde ich nicht ausreichend bezahlt.
**12.**	Die Klinik und das UKM bieten mir optimale Möglichkeiten, mich weiterzuentwickeln und in meinem Beruf Karriere zu machen.	1 2 3 4 5 6 7 8 9 10	Die Klinik und das UKM sehe ich als Sackgasse, in der ich mich beruflich nicht weiter entwickeln kann.
**13.**	Nach meiner Erfahrung ist die Arbeitsbelastung in der Klinik nicht zu hoch, und das wird auch so bleiben.	1 2 3 4 5 6 7 8 9 10	Nach meiner Erfahrung ist die Arbeitsbelastung unerträglich und es wird in Zukunft nicht besser.

GEFÖRDERT VOM
Bundesministerium für Bildung und Forschung

- Für wie wahrscheinlich halten Sie es, dass Sie in fünf Jahren noch an der Klinik tätig sind? (0 = sehr unwahrscheinlich; 10 = äußerst wahrscheinlich)

0	1	2	3	4	5	6	7	8	9	10
☐	☐	☐	☐	☐	☐	☐	☐	☐	☐	☐

- Was sind die Gründe für Ihre Einschätzung?

GEFÖRDERT VOM
Bundesministerium für Bildung und Forschung

„Repertory Grid“

- Verfahren zur Erfassung von individuellen Einstellungen
- Basis ist die Theorie der persönlichen Konstrukte nach George A. Kelly
  - Persönliche Konstrukte sind die Art und Weise, wie Menschen ihre Welt sehen
  - Menschen beschreiben ihre Umwelt meist durch den Vergleich von Elementen
- Ablauf
  - Vergleich von zwei Elementen über Gemeinsamkeiten oder Unterschiede
  - Beschreibung von Gemeinsamkeiten und Unterschieden
  - Einordnung aller Elemente auf einer Skala
- Beispiel „Automarken“

  1. Die Elemente weisen mehr Unterschiede als Gemeinsamkeiten auf:

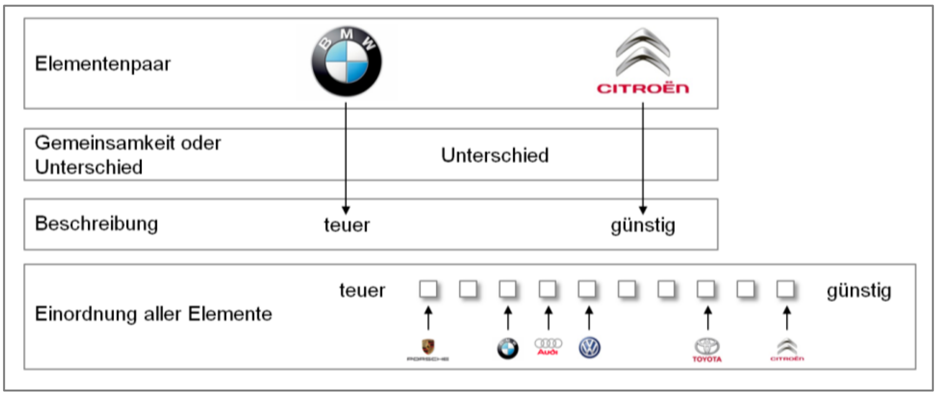

  2. Die Elemente weisen mehr Gemeinsamkeiten als Unterschiede auf:

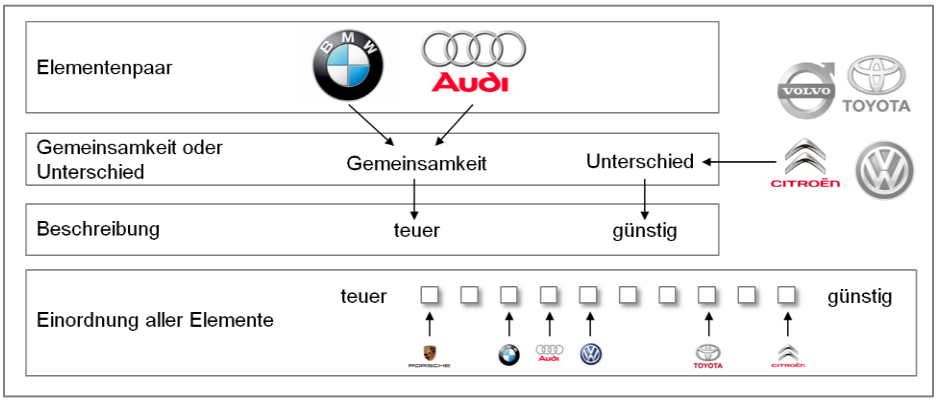

- Bearbeitung von „Repertory Grids“ für die Elemente:

  1. das Ärzteteam – das Pflegeteam
  2. die Klinik heute – die Klinik in 5 Jahren
  3. UKM-Verwaltung – Klinikverwaltung
  4. das UKM heute – das UKM in 5 Jahren
  5. das UKM heute – die Klinik heute
  6. die Klinik in 5 Jahren – das UKM in 5 Jahren

GEFÖRDERT VOM
Bundesministerium für Bildung und Forschung

- Erläuterungen der Elemente:
  - das Ärzteteam = Team aus Assistenz-, Fach- und Oberärzten
  - das Pflegeteam = Team aus Direktion, (Bereichs-)Leitung und Mitarbeitern der Pflege
  - die Klinik heute = Gesamtbild/Arbeitsumfeld heute
  - die Klinik in 5 Jahren = realistisches Gesamtbild/Arbeitsumfeld in 5 Jahren, kein Wunschbild
  - Verwaltung des UKM = Administrative und planerische Tätigkeiten (z.B. Lohn- und Gehaltsabrechnung, OP-Management etc.)
  - Verwaltung der Klinik = Administrative und planerische Tätigkeiten (z.B. Personaleinsatzplanung, Urlaubsvergabe etc.)
  - das UKM heute = Gesamtbild/Arbeitsumfeld heute
  - das UKM in 5 Jahren = realistisches Gesamtbild/Arbeitsumfeld in fünf Jahren, kein Wunschbild

Translation of the German original

Invitation to an interview

Dear Lady, Dear Sir,

we write to you from the office of the joint project PhysicianPlus. As already announced, we would be pleased if you could be available to us in the course of the project in the next few weeks for a 60-minute one-on-one conversation.

We would like to ask you about your experiences and wishes regarding your everyday work. On the one hand, the interview is based on a guideline, on the other hand, we will conduct part of the interview with software support. By participating in this interview, you are creating an important basis for further project work and actively contributing to the success of the project.

For the coordination of the first interviews, we have created an overview of the dates and would like to ask you to enter your desired date stating your name. All entries are anonymous and are not visible to your colleagues. We will then send you an appointment confirmation by e-mail. If none of the suggested dates appeal to you, please let us know a possible alternative date.

At this point, we would like to emphasize once again that the results of the individual discussions are treated strictly confidentially and used exclusively within the framework of the PhysicianPlus project. The evaluation will only be carried out summarily, which means that no conclusions can be drawn about your person.

If you have any questions about the project or the interviews, please do not hesitate to contact us. An overview of project goals and procedures can be found attached.

We look forward to your cooperation and the conversation with you!

Sincerely,

Joint project PhysicianPlus
- Conversation Guide -

Interlocutors

Function/Position:
An of the clinic since:
In current position since:

Interviewer:

Date/Time:

Special features of the clinic

- What do you experience as a special strength of your clinic?
- Where do you see weak points within your clinic?
- Would you recommend a friend or colleague to work at your clinic?
(0 = very unlikely; 10 = extremely likely)

0	1	2	3	4	5	6	7	8	9	10
☐	☐	☐	☐	☐	☐	☐	☐	☐	☐	☐

- Would you recommend further education and training at your clinic to a friend or colleague? (0 = very unlikely; 10 = extremely likely)

0	1	2	3	4	5	6	7	8	9	10
☐	☐	☐	☐	☐	☐	☐	☐	☐	☐	☐

- How do you justify your recommendations?
- How do you experience your personal daily work at the clinic? (1 = statement on the left, 10 = statement on the right or a value in between)

**1.**	For my work I get enough appreciation and support from...		My work is little appreciated and unnecessarily criticized by…
1.1	…Colleagues.	1 2 3 4 5 6 7 8 9 10	…Colleagues.
1.2	…Superiors.	1 2 3 4 5 6 7 8 9 10	…Superiors.
**2.**	My superiors know my personal goals and take them into account as far as possible.	1 2 3 4 5 6 7 8 9 10	My personal goals are neither perceived nor taken into account by my superiors.

**3.**	You can always rely on promises made by the clinic and superiors.	1 2 3 4 5 6 7 8 9 10	Promises cannot be trusted because they are not kept.
**4.**	When and where I have to work, I can plan for the long term.	1 2 3 4 5 6 7 8 9 10	Changing places and times of work cannot be planned for me.
**5.**	I am informed in a timely and sufficient manner about plans and decisions that affect my work..	1 2 3 4 5 6 7 8 9 10	I am often not informed in time and sufficiently about plans and decisions that are important to me.
**6.**	Decisions that affect my work as well as the decision-making process are easy for me to understand.	1 2 3 4 5 6 7 8 9 10	I often can't understand decisions and decision-making processes.
**7.**	Within the given framework, I can decide for myself how I do my work.	1 2 3 4 5 6 7 8 9 10	I have no room for manoeuvre in decision-making and feel that I am being thwarted by specifications in my work.
**8.**	The work offers many challenges, but I never feel overwhelmed.	1 2 3 4 5 6 7 8 9 10	I feel overwhelmed by the demands of my work.
**9.**	In the clinic, I do meaningful work that benefits society.	1 2 3 4 5 6 7 8 9 10	I do pointless work that is of no use to anyone.
**10.**	In the clinic I find working conditions that are important to me and that I could not find anywhere else.	1 2 3 4 5 6 7 8 9 10	I might as well work in another hospital.
**11.**	I am paid fairly and appropriately for my work.	1 2 3 4 5 6 7 8 9 10	I don't get paid enough for the work I do.
**12.**	The clinic offersme optimal opportunities to develop further and to make a career in my profession.	1 2 3 4 5 6 7 8 9 10	I see the clinic as a dead end in which I cannot develop professionally.
**13.**	In my experience, the workload in the clinic is not too high, and it will remain so..	1 2 3 4 5 6 7 8 9 10	In my experience, the workload is unbearable and it won't get better in the future.

- How likely do you think it is that you will still be working at the clinic in five years' time?
  (0 = very unlikely; 10 = extremely likely)

0	1	2	3	4	5	6	7	8	9	10
☐	☐	☐	☐	☐	☐	☐	☐	☐	☐	☐

- What are the reasons for your assessment?

GEFÖRDERT VOM
Bundesministerium für Bildung und Forschung

„Repertory Grid“

1. Procedure for recording individual settings
2. The basis is the theory of personal constructs according to George A. Kelly
   1. Personal constructs are the way people see their world
   2. People usually describe their environment by comparing elements
3. Expiration
   1. Comparison of two elements via similarities or differences
   2. Description of similarities and differences
   3. Classification of all elements on a scale
4. Example "Car Brands"

   1. The elements have more differences than similarities:

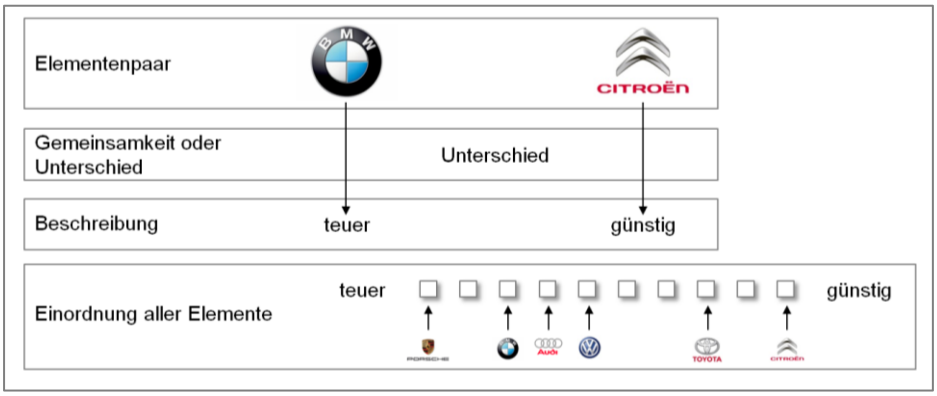

Text in image:

pair of elements <BMW>, <Citroen>

Commonality or difference <Difference>

Description <expensive>,< auspicious >

Classification of all elements <expensive > □ □ □ □ □ □ □ □ □ □ <auspicious>

2. The elements have more similarities than differences:

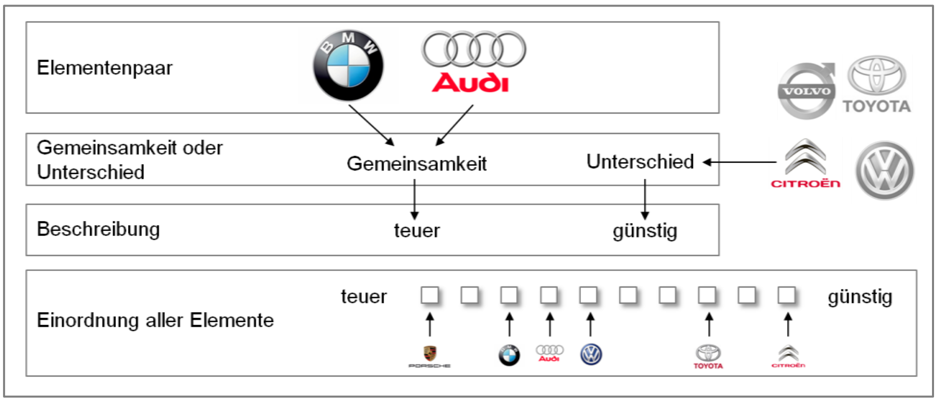

Text in image:

pair of elements <BMW>, <Audi>

Commonality or difference <Commonality>, <Difference>

Description <expensive>,< auspicious >

Classification of all elements <expensive > □ □ □ □ □ □ □ □ □ □ <auspicious>

- Generating "Repertory Grids" for the elements:

1. the medical team – the nursing team
2. the clinic today – the clinic in 5 years
3. University Hospital Administration – Clinic Administration
4. the University Hospital today – the University Hospital in 5 years
5. the University Hospital today – the clinic today
6. the clinic in 5 years – the University Hospital in 5 years

- Explanations of the elements:
    1. the medical team = team of assistants, specialists and senior physicians
    2. the nursing team = team of management, (area) management and nursing staff
    3. the clinic today = overall picture/working environment today
    4. the clinic in 5 years = realistic overall picture/working environment in 5 years, no dream image
    5. Administration of the University Hospital = Administrative and planning activities (e.g. payroll, OP management, etc.)
    6. Administration of the clinic = administrative and planning activities (e.g. personnel deployment planning, vacation assignment, etc.)
    7. the University Hospital today = overall picture/working environment today
    8. the University Hospital in 5 years = realistic overall picture/working environment in five years, no dream image

GEFÖRDERT VOM
Bundesministerium für Bildung und Forschung
