# Supplementary material for: Individual perspectives and mental maps of working conditions and intention to stay of physicians in academic medicine
Source: Front Psychol. 2023 May 12;14:1106501. doi: 10.3389/fpsyg.2023.1106501 (PMC10213555; doi:10.3389/fpsyg.2023.1106501)
Supplement: Supplementary Data Sheet 3 — Interview Result Protocol (German Original plus English Translation). [file Data_Sheet_3.PDF]

Ergebnisprotokoll:

„Verbundprojekt FacharztPlus“ – Facharztinterview

**Datum**	dd.mm.yyyy
**Uhrzeit**	hh:mm – hh:mm
**Teilnehmer/innen**	<name> Subject 1 (main interview)
**Protokollant**	<name>

TOP	Ihre Beiträge
**1**	**Persönliche Vorstellung**
1.1	Facharzt mit Spezialisierung in spez. Schmerztherapie<br>• Im UKM seit 6 Jahren<br>• Facharzt seit 6 Jahren<br>• Vor allem Betreuung der sog. Aussenbereiche (Psychiatrie, Kinderonkologie, Dermatologie, MRT etc.)
**2**	**Besonderheiten Klinik**
2.1	Stärken:<br>• Gute Kernarbeitszeiten (jedoch derzeit in Veränderung)<br>• Guter Zugang zu Ansprechpartnern (wenn man sich auskennt)<br>• Größe und Kompetenz in der Patientenversorgung (stolz darauf „state oft the art“ zu arbeiten)<br>• Bestrebe auf individuelle Wünsche einzugehen, „Nischen“ zu schaffen (von denen es aber zu wenig gibt)
2.2	Schwächen:<br>• Reputation von Fachärzten: Anästhesie nicht als „Dienstleister“ sondern als vollwertige Ärzte „mit Rückgrat“ positionieren.<br>• Assistenten in dieser Haltung stärken, um Kernarbeitszeit zu 100% zu nutze.<br>• Wechselseitiges Verständnis für medizinisch verursachte Verzögerungen sowohl bei der Operation als auch bei der Ein- bzw. Ausleitung der Anästhesie.<br>• Notfälle müssen in entstehenden Lücken eingepflegt, was durch vielen OP-Säle und Größe möglich ist<br>• Ziel sollte der unbedingte Erhalt der normalen Arbeitszeit sein.<br>• Zeit von Arbeitsende bis Arbeitswiederbeginn muss lang genug zur Erholung sein<br>• Aufstiegschancen müssten verbessert und Seniorität anerkannt werden (z. B. Beförderung zum Oberarzt / Funktionsoberarzt)<br>• Durch teilweise nicht optimale Nutzung der Kernarbeitszeiten (z. B. Warten auf Operateure) rutscht elektives OP-Programm außerhalb der Kernarbeitszeiten in den Abend<br>• Wenig Austausch zwischen der (Fach-)Ärzt/inn/en

<table>
<tr><td>2.3</td><td>Ideen zur Verbesserung:
Gleichwertigkeit der Leistung in KV und F+L sicherstellen: Leistung in der KV schafft erst den Freiraum für Forschung
Bessere Nutzung der Kernarbeitszeiten insbes. im OP („D-Zug auf Schiene statt Trecker auf Acker“)
Mehr Austausch ermöglichen
Gemeinsame Ideen entwickeln um die Corporate Identity zu fördern
Fach- und hierachieübergreifend klarstellen, dass Loyalität zum Arbeitgeber für Erhalt der Arbeitsplätze wichtig ist</td></tr>
<tr><td>2.4</td><td>Einschätzung einer Weiterempfehlung (0 = sehr unwahrscheinlich bis 10 = äußerst wahrscheinlich):
Empfehlung Arbeit: 8
Empfehlung Training: 10</td></tr>
<tr><td>2.5</td><td>Hauptgründe für Verlassen der Klinik:
Mangelnde Aufstiegschancen
Nachteilige Veränderung der Persönlichkeit unter Druck
Bei anstrengenden Diensten (insbes. „Turmdienst“) wenig Aussicht auf Veränderung / Verbesserung
Keine „Nische“ als eigener Gestaltungsfreiraum
Gelegentlich Vermutung das es anderswo „besser“ sei
Depressive Entwicklung
Finanzielle Begrenztheit
Abwerbung durch bessere Bezahlung</td></tr>
<tr><td>2.6</td><td>Ideen zur längeren Facharztbindung:
Entstehende Nischen (wachsende Außenbereiche, Ambulatorium) nutzen, um „Nischen“ und Gestaltungsfreiräume zu schaffen
Für Ältere (z. B. nach Vorbild Opel / Daimler) „Ruheräume“ in weniger anstrengenden Diensten und Freiräume ermöglichen
Arbeitsleistung nicht nur über „mehr Arbeitszeit“ sondern vor allem über „bessere Zeitnutzung“ steigern: besser getaktete Wechselzeiten zwischen den OPs
Weniger Aufgaben außerhalb, mehr in der KV
Ärzt/inn/e/n schon als Assistenten mehr an Verantwortung geben und Gestaltungsmöglichkeiten bieten
Nicht immer auf volle Arbeitszeit (oder Mehrarbeit) bestehen, sondern nach Beendigung der Aufgaben konsequent in den Feierabend schicken sozusagen als Belohnung
selbstbestimmtes Arbeiten steigert Berufszufriedenheit
möglichst flache Hierarchie umsetzen
Finanzielle Anreize bieten: Toparbeit = Topbezahlung</td></tr>
<tr><td>3</td><td>Unternehmenskultur</td></tr>
<tr><td>3.1</td><td>Zusammenarbeit
Stellungsunterschiede werden manchmal ausgespielt (Arzt „auflaufen“ lassen, Krankenschwester „zappeln“ lassen)
Mehr Ergebnisorientierung und Einsatzbereitschaft statt einer manchmal noch vorherrschenden „Landesbedienstetenkultur“
Loyalität zum UKM sollte gefördert werden
Facharzt ist oft „Motivator“ und „Erklärer“ (z. B. das Wartezeiten und geringe Nutzungszeiten teurer Geräte Kosten verursachen)
Wenig Kontakt zur Verwaltung, dann meist über „InfoPoint“
Zusammenarbeit mit ärztlichen Kolleg/inn/en gut, wenn man selbst mit gestalten kann, in anderen Bereichen (z. B. Chirurgie) manchmal nicht so gut</td></tr>
</table>

**4**	**Organisation / Führung**
4.1	Gut funktionierende Prozesse:<br>• Einteilung der Dienste nach einem internen System (sog. „Bröckelmann-System“) funktioniert ganz gut<br>• Urlaub ist noch im Umbruch und noch nicht gut: Interne Kommission setzt falsches Signal, weil Ärzte noch mehr in Planung / Verwaltung hinein gezogen werden)<br>• Externe Fortbildung eher selten, da oft Verweis auf interne Weiterbildung
4.2	Verbesserungsmöglichkeiten:<br>• Möglichst viel ärztliches Personal in der Krankenversorgung (und nicht z. B. Planung) einsetzen<br>• Verwaltung bzw. Verwaltungsangestellte sollten Ärzte entlasten<br>• Ärzte zurückholen in die originäre ärztliche Tätigkeit, Verwaltungsaufgaben konsequent von der Verwaltung einfordern<br>• Kernarbeitszeiten mit entsprechender Planung und für alle verbindliche Umsetzung optimal nutzen
4.3	Ressourcen:<br>• Sind zur Erfüllung der Aufgaben ausreichend
4.4	Formen der Personalführung und Verbesserungspotenziale:<br>• Mitarbeitergespräch als Karrieregespräch in persönlicher 1:1 Atmosphäre einmal jährlich führen<br>• Wenn möglich Abstimmungen in Teams geben (z. B. Planung, wer wann an externen Fortbildungen teilnehmen kann)<br>• Paritätische, hierarchiearme Strukturen einführen
**5**	**Personalentwicklung**
5.1	Bewertung eigene Einarbeitung:<br>• War auch wg. Vorerfahrung am UKM gut<br>• Tutorensystem ist etabliert und funktioniert
5.2	Einarbeitung ausländischer Mitarbeiter/innen:<br>• In Einzelfällen werden ausländische oder ältere Kollegen „belächelt“, weil unterstellt wird, dass sie ggf. nicht dieselbe Leistung bringen können wie bereits vorhandene: Hier fehlt es an persönlicher Unterstützung und Wertschätzung
5.3	Verbesserungsmöglichkeiten:<br>• Persönliche Wertschätzung und Unterstützung deutlich machen<br>• Attraktive Fortbildungsmöglichkeit gerade auch zur Bindung jüngerer Kollegen (z. B. Fortbildung an attraktivem Urlaubsort)
**6**	**Perspektiven für FacharztPlus**
6.1	Ziele des Projekts und Informationswunsch:<br>• Projekt ist gut und signalisiert bereits Wertschätzung für die Fachärzt/inn/e/n<br>• Nicht nur Prozesse optimieren, sondern auch „Wohlfühlfaktor“ berücksichtigen (persönliche Gespräche, Nischen, attraktive Fortbildungs- und Aufstiegsmöglichkeiten)<br>• Information per Newsletter und „Montagsvorlesung“<br><br>Wahrscheinlichkeit zu bleiben (0 = sehr unwahrscheinlich bis 10 = äußerst wahrscheinlich):<br>• 8

6.2	Kommentare/Hinweise zu FacharztPlus:<br>• „Was kann man zum Erhalt der Gesundheit der Fachärzte beitragen?“<br>• Freiwillige Analyse von Krankheitsdiagnosen der Mitarbeiter, um frühzeitig auf Fehlentwicklungen reagieren zu können

Einwilligungserklärung

Hiermit bestätige ich, dass ich einverstanden bin, dass die Ergebnisse meines Interviews im FacharztPlus-Projekt in anonymisierter Form verwendet werden dürfen.
Ich habe die Information zum Projekt und die Erklärung zum Datenschutz erhalten, gelesen und verstanden und habe keine weiteren Fragen.
Ich bin mir meiner Rechte als Proband bei dieser Studie bewusst.

____
Name und Vorname in Druckbuchstaben

________________________________ ________________________________
Ort und Datum Unterschrift

Translation of the German original

Main Interview Subject 1

TOP	Your contributions
**1**	**Personal introduction**
1.1	Specialist with specialization in spec. Pain<br>1. In the UKM for 6 years<br>2. Specialist for 6 years<br>• Above all, care of the so-called outdoor areas (psychiatry, pediatric oncology, dermatology, MRI, etc.)
**2**	**Special features clinic**
2.1	Strengths:<br>1. Good core working hours (but currently changing)<br>2. Good access to contact persons (if you are familiar with each other)<br>3. Size and competence in patient care (proud to work "state of the art")<br>• Strive to respond to individual desires, to create "niches" (of which there are too few)
2.2	Weaknesses:<br>1. Reputation of specialists: Position anesthesia not as a "service provider" but as full-fledged doctors "with backbone".<br>2. Strengthen assistants in this attitude in order to use core working time 100%.<br>3. Mutual understanding of medically caused delays both during surgery and in the introduction or discharge of anesthesia.<br>4. Emergencies have to be filled into emerging gaps, which is made possible by many operating theatres and sizes<br>5. The aim should be the unconditional preservation of normal working hours.<br>6. Time from the end of work to the start of work must be long enough to recover<br>7. Opportunities for advancement would have to be improved and seniority recognised (e.B. promotion to senior physician / functional senior physician)<br>8. Due to partly not optimal use of the core working hours (e.B. waiting for surgeons), elective surgical program slips into the evening outside the core working hours<br>• Little exchange between the (specialist) doctors

2.3	Ideas for improvement:<br>• Ensuring equivalence of performance in CT and F+L: Performance in CT creates the scope for research<br>• Better use of core working hours, in example. in the operating room ("train on rail instead of tractor on field")<br>• Enable more exchange<br>• Developing common ideas to promote corporate identity<br>• Make it clear across disciplines and authorities that loyalty to the employer is important for the preservation of jobs<br>•
2.4	Assessment of a recommendation (0 = very unlikely to 10 = extremely likely):<br>• Recommendation work: 8<br>• Recommendation Training: 10<br>•
2.5	Main reasons for leaving the clinic:<br>1. Lack of opportunities for advancement<br>2. Disadvantageous change in personality under pressure<br>3. In the case of strenuous services (especially in the case of strenuous services). "Tower service") little prospect of change / improvement<br>4. No "niche" as your own creative freedom<br>5. Occasional assumption that it is "better" elsewhere<br>6. Depressive development<br>7. Financial limitations<br>8. Poaching through better pay
2.6	Ideas for longer retention of doctors:<br>1. Use emerging niches (growing outdoor areas, outpatient clinic) to create "niches" and design freedom<br>2. For the elderly (e.B. following the example of Opel / Daimler) enable "rest rooms" in less strenuous services and free spaces<br>3. Increase work performance not only through "more working time" but above all through "better use of time": better timed change times between the operating operations<br>4. Fewer tasks outside, more in KV<br>5. Give doctors more responsibility and design options even as assistants<br>6. Do not always insist on full working hours (or extra work), but consistently send them into the evening after completion of the tasks, so to speak, as a reward<br>7. Self-determined work increases job satisfaction<br>8. Implement the flatest possible hierarchie<br>• Offering financial incentives: Top work = top pay
**3**	**Corporate Culture**
3.1	Cooperation<br>1. Differences in position are sometimes played out (let the doctor "run up", let the nurse "fidget")<br>2. More focus on results and commitment instead of a sometimes still predominant "state employee culture"<br>3. Loyalty to the UKM should be encouraged<br>4. Specialist is often a "motivator" and "explainer" (e.g. that waiting times and short usage times of expensive devices cause costs)<br>5. Little contact with the administration, then usually via "InfoPoint"<br>• Cooperation with medical colleagues is good, if you can help shape things yourself, in other areas (e.B. surgery) sometimes not so well
**4**	**Management / Leadership**

4.1	Well-functioning processes:<br>1. Classification of services according to an internal system (so-called "Bröckelmann system") works quite well<br>2. Holidays are still in a state of upheaval and not yet good: Internal commission sends the wrong signal because doctors are drawn even more into planning / administration)<br>• External training is rather rare, as reference is often made to internal training
4.2	Improvements:<br>1. Use as much medical staff as possible in health care (and not e.g. planning)<br>2. Administration or administrative employees should relieve doctors<br>3. Bringing doctors back into the original medical activity, consistently demanding administrative tasks from the administration<br>• Optimal use of core working hours with appropriate planning and binding implementation for all
4.3	Resources:<br>• Are sufficient to perform the tasks
4.4	Forms of personnel management and potential for improvement:<br>1. Conduct an employee appraisal as a career interview in a personal 1:1 atmosphere once a year<br>2. If possible, give coordination in teams (e.g. planning who can participate in external training courses and when)<br>• Introducing parity- and low-hierarchy structures
**5**	**Staff development**
5.1	Evaluation of own onboarding:<br>1. Was also wg. previous experience at the clinic good<br>• Tutor system is established and works
5.2	Onboarding of foreign employees:<br>• In individual cases, foreign or older colleagues are "ridiculed" because it is assumed that they may not be able to perform as already existing ones: Here there is a lack of personal support and appreciation
5.3	Improvements:<br>1. Making personal appreciation and support clear<br>2. Attractive training opportunities, especially for retaining younger colleagues (e.g. further training at an attractive holiday location)
**6**	**Perspectives for PhysicianPlus**
6.1	Objectives of the project and request for information:<br>1. Project is good and already signals appreciation for the specialists<br>2. Not only optimize processes, but also take into account the "feel-good factor" (personal conversations, niches, attractive training and promotion opportunities)<br>3. Information via newsletter and "Monday lecture"<br><br>Probability of staying (0 = very unlikely to 10 = extremely likely):<br>• 8
6.2	Comments/Notes on PhysicianPlus:<br>1. "What can be contributed to maintaining the health of specialists?"<br>• Voluntary analysis of employees' disease diagnoses in order to be able to react to undesirable developments at an early stage

Consent

I hereby confirm that I agree that the results of my interview in the FacharztPlusproject may be used in anonymous form.
I have received, read and understood the information about the project and the declaration on data protection and have no further questions.
I am aware of my rights as a subject in this study.

____
Surname and first name in block letters

__________________________________ ______________________________
Place and date Signature
