# Supplementary material for: Individual perspectives and mental maps of working conditions and intention to stay of physicians in academic medicine
Source: Front Psychol. 2023 May 12;14:1106501. doi: 10.3389/fpsyg.2023.1106501 (PMC10213555; doi:10.3389/fpsyg.2023.1106501)
Supplement: Supplementary Data Sheet 5 — Supplementary Appendices. [file Data_Sheet_5.pdf]

Supplementary Appendices

Appendix 1: Interview guide preliminary and main interviews

A1.1 Interview Guide, Pre-Study
(Translation of the German original, see supplementary file #1)

Dear physicians,

the first phase of our *Project PhysicianPlus* is running to the fullest. As already announced, we want to achieve with the joint project that your work is even more oriented to your individual needs, competencies and goals.

Central to the success of this project is therefore above all your opinion and active cooperation. We would like to create the basis for this through an initial conversation, in which we would like to obtain your opinions, suggestions and wishes for the *PhysicianPlus* project and get to know you personally. Topics include the special features of the UKM (University Hospital Muenster) and your clinic in terms of organization and leadership.

We would be very pleased if you would be available to us in the course of the next few weeks for a 30-45 minutes one-on-one conversation. For a better coordination of the discussions, we have created an overview of the dates and would like to ask you to enter your desired date stating your name. All entries are anonymous and are not visible to your colleagues. We will then send you a separate appointment confirmation by email.

At this point, we would like to emphasize once again that the results of the discussions are treated as strictly confidential and used exclusively for the *PhysicianPlus* project. The evaluation will only be carried out summarily, which means that no conclusions can be drawn about your person. The minutes made after the interview will be sent to you and can make comments on them. The results will only be used after your written approval.

We look forward to your cooperation and the discussions with you!
Sincerely,
[…]

Joint project PhysicianPlus
- Conversation Guide -

Interlocutors
Function:
Hospital since:
Specialist since:

Interviewer:
Date/Time:

Personal introduction

- What are your tasks within the clinic?

Special features Clinic

- What do you experience as a special strength of your clinic?
- Where do you see vulnerabilities?
- Do you have any suggestions on how to counteract the weaknesses?
- Would you recommend a friend or colleague from another clinic to work as a specialist in your clinic? (0 = very unlikely; 10 = extremely likely)

0	1	2	3	4	5	6	7	8	9	10
☐	☐	☐	☐	☐	☐	☐	☐	☐	☐	☐

- Would you recommend a friend or doctor from another clinic to complete further training as a specialist in your clinic?
  (0 = very unlikely; 10 = extremely likely)

0	1	2	3	4	5	6	7	8	9	10
☐	☐	☐	☐	☐	☐	☐	☐	☐	☐	☐

- Do you know any reasons why specialists have left the clinic?

- What would have to be done so that specialists continue to work at the clinic for a longer period of time (2-5 years) after completing their further training?

- How could this be achieved?

How do you experience the cooperation with the different employee groups (nurses, administration, technical service)?

- How do you assess the cooperation between doctors at all hierarchical levels?

Organization and leadership culture

- How do you assess the personnel planning processes at your clinic (planning of services, holidays, additional qualifications)?
- What could be done better from your point of view?
- Are there enough resources at your clinic to successfully cope with your tasks (personnel, materials, equipment, financial resources)?
- What regular forms of personnel management and support do you experience (e.B feedback, appraisal or development interview with the supervisor)?
- What would you like to improve or supplement?

Staff development

- How did you experience your own induction at the clinic? What would you possibly improve?
  From your point of view, how good is the induction of non-German-speaking colleagues? What should be improved?

Perspective of specialists in the clinic

- Do you like the goals of the 'FacharztPlus' project?
- Which goals should be supplemented or adapted if necessary?
- How would you like to be informed about project results?

- Do you think that you will still work for this clinic, 5 years ago?
  (0 = very unlikely; 10 = extremely likely)

0	1	2	3	4	5	6	7	8	9	10
☐	☐	☐	☐	☐	☐	☐	☐	☐	☐	☐

- At the end of our conversation, do you have any comments or hints about the Project PhysicianPlus?

A1.2 Main Interviews with Repertory Grids

(Translation of the German original, see supplementary file #2)

Invitation to an interview

Dear Lady, Dear Sir,
we write to you from the office of the joint project PhysicianPlus. As already announced, we would be pleased if you could be available to us in the course of the project in the next few weeks for a 60-minute one-on-one conversation.

We would like to ask you about your experiences and wishes regarding your everyday work. On the one hand, the interview is based on a guideline, on the other hand, we will conduct part of the interview with software support. By participating in this interview, you are creating an important basis for further project work and actively contributing to the success of the project.

For the coordination of the first interviews, we have created an overview of the dates and would like to ask you to enter your desired date stating your name. All entries are anonymous and are not visible to your colleagues. We will then send you an appointment confirmation by e-mail. If none of the suggested dates appeal to you, please let us know a possible alternative date.

At this point, we would like to emphasize once again that the results of the individual discussions are treated strictly confidentially and used exclusively within the framework of the PhysicianPlus project. The evaluation will only be carried out summarily, which means that no conclusions can be drawn about your person.

If you have any questions about the project or the interviews, please do not hesitate to contact us. An overview of project goals and procedures can be found attached.

We look forward to your cooperation and the conversation with you!

Sincerely,
[…]

Joint project PhysicianPlus
- Conversation Guide -

Interlocutors
Function/Position:
An of the clinic since:
In current position since:

Interviewer:
Date/Time:

Special features of the clinic

- What do you experience as a special strength of your clinic?
- Where do you see weak points within your clinic?

- Would you recommend a friend or colleague to work at your clinic?

(0 = very unlikely; 10 = extremely likely)

0	1	2	3	4	5	6	7	8	9	10
☐	☐	☐	☐	☐	☐	☐	☐	☐	☐	☐

- Would you recommend further education and training at your clinic to a friend or colleague? (0 = very unlikely; 10 = extremely likely)

0	1	2	3	4	5	6	7	8	9	10
☐	☐	☐	☐	☐	☐	☐	☐	☐	☐	☐

- How do you justify your recommendations?

- How do you experience your personal daily work at the clinic? (1 = statement on the left, 10 = statement on the right or a value in between)

**1.**	For my work I get enough appreciation and support from...		My work is little appreciated and unnecessarily criticized by…
1.1	…Colleagues.	1 2 3 4 5 6 7 8 9 10	…Colleagues.
1.2	…Superiors.	1 2 3 4 5 6 7 8 9 10	…Superiors.
**2.**	My superiors know my personal goals and take them into account as far as possible.	1 2 3 4 5 6 7 8 9 10	My personal goals are neither perceived nor taken into account by my superiors.
**3.**	You can always rely on promises made by the clinic and superiors.	1 2 3 4 5 6 7 8 9 10	Promises cannot be trusted because they are not kept.
**4.**	When and where I have to work, I can plan for the long term.	1 2 3 4 5 6 7 8 9 10	Changing places and times of work cannot be planned for me.
**5.**	I am informed in a timely and sufficient manner about plans and decisions that affect my work..	1 2 3 4 5 6 7 8 9 10	I am often not informed in time and sufficiently about plans and decisions that are important to me.
**6.**	Decisions that affect my work as well as the decision-making process are easy for me to understand.	1 2 3 4 5 6 7 8 9 10	I often can't understand decisions and decision-making processes.
**7.**	Within the given framework, I can decide for myself how I do my work.	1 2 3 4 5 6 7 8 9 10	I have no room for manoeuvre in decision-making and feel that I am being thwarted by specifications in my work.
**8.**	The work offers many challenges, but I never feel overwhelmed.	1 2 3 4 5 6 7 8 9 10	I feel overwhelmed by the demands of my work.
**9.**	In the clinic, I do meaningful work that benefits society.	1 2 3 4 5 6 7 8 9 10	I do pointless work that is of no use to anyone.
**10.**	In the clinic I find working conditions that are important to me and that I could not find anywhere else.	1 2 3 4 5 6 7 8 9 10	I might as well work in another hospital.
**11.**	I am paid fairly and appropriately for my work.	1 2 3 4 5 6 7 8 9 10	I don't get paid enough for the work I do.
**12.**	The clinic offersme optimal opportunities to develop further and to make a career in my profession.	1 2 3 4 5 6 7 8 9 10	I see the clinic as a dead end in which I cannot develop professionally.
**13.**	In my experience, the workload in the clinic is not too high, and it will remain so..	1 2 3 4 5 6 7 8 9 10	In my experience, the workload is unbearable and it won't get better in the future.

- How likely do you think it is that you will still be working at the clinic in five years' time? (0 = very unlikely; 10 = extremely likely)

0	1	2	3	4	5	6	7	8	9	10
☐	☐	☐	☐	☐	☐	☐	☐	☐	☐	☐

- What are the reasons for your assessment?

Repertory Grids

1. Procedure for recording individual settings

2. The basis is the theory of personal constructs according to George A. Kelly
   - Personal constructs are the way people see their world
   - People usually describe their environment by comparing elements

3. Expiration
   - Comparison of two elements via similarities or differences
   - Description of similarities and differences
   - Classification of all elements on a scale

4. Example "Car Brands"
1. The elements have more differences than similarities:

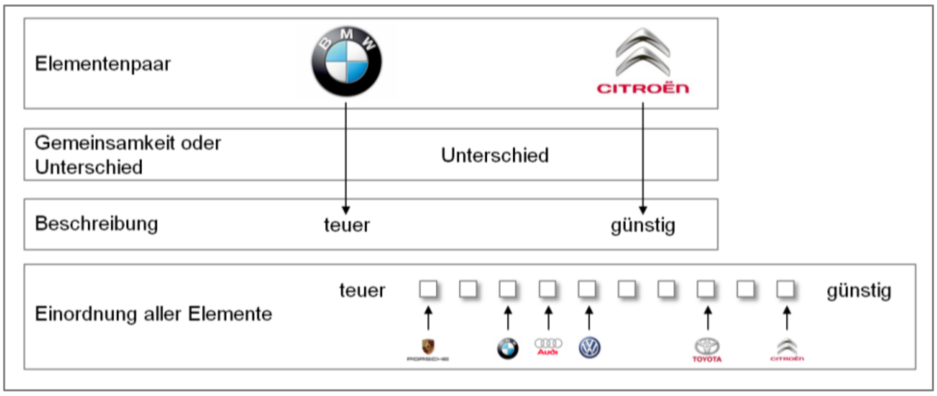

Text in image:
- pair of elements <BMW>, <Citroen>
- Commonality or difference <Difference>
- Description <expensive>,< auspicious >

Classification of all elements <expensive > ☐☐☐☐☐☐☐☐☐☐ <auspicious>

2. The elements have more similarities than differences:

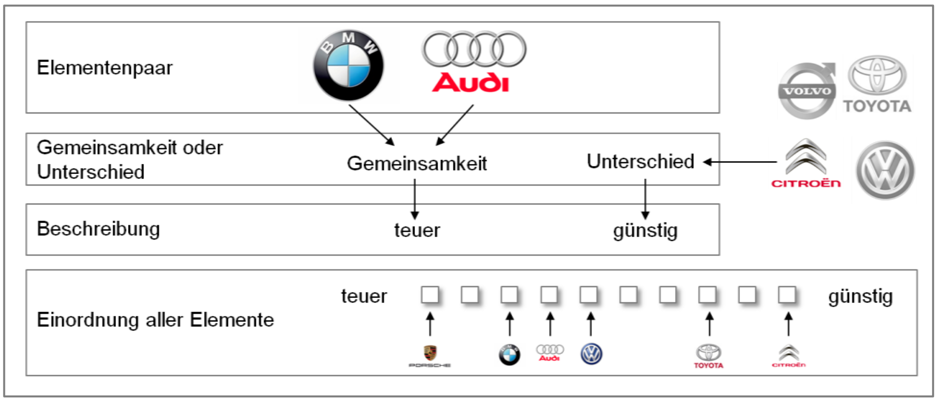

Text in image:
- pair of elements <BMW>, <Audi>
- Commonality or difference <Commonality>, <Difference>
- Description <expensive>,< auspicious >

Classification of all elements <expensive > ☐☐☐☐☐☐☐☐☐☐ <auspicious>

- Generating "Repertory Grids" for the elements:
    1. the medical team – the nursing team
    2. the clinic today – the clinic in 5 years
    3. University Hospital Administration – Clinic Administration
    4. the University Hospital today – the University Hospital in 5 years
    5. the University Hospital today – the clinic today
    6. the clinic in 5 years – the University Hospital in 5 years

- Explanations of the elements:
    1. the medical team = team of assistants, specialists and senior physicians
    2. the nursing team = team of management, (area) management and nursing staff
    3. the clinic today = overall picture/working environment today
    4. the clinic in 5 years = realistic overall picture/working environment in 5 years, <u>no</u> dream image
    5. Administration of the University Hospital = Administrative and planning activities (e.g. payroll, OP management, etc.)
    6. Administration of the clinic = administrative and planning activities (e.g. personnel deployment planning, vacation assignment, etc.)
    7. the University Hospital today = overall picture/working environment today
    8. the University Hospital in 5 years = realistic overall picture/working environment in five years, <u>no</u> dream image

Appendix 2:

Intercorrelation of sum of all statements about a topic (upper triangle) and difference of positive and negative statements (lower triangle)

	Training	Duty	Vacation	Culture	Capacity	Resources	Leader-ship	Perfor-mance	On-boarding	Work env.	Coope-ration	Tech. serv.	Admini-stration	Work -Life	Super-vision	Salary	Career	Work flex.	Coop. nurses	Work. hours
Training	1	-.016	-.063	.120	-.046	.094	.234	-.140	.113	.217	.138	.032	.018	-.094	-.074	-.211	.000	.093	-.059	.031
Duty Sched.	-.098	1	.228	.055	.229	.088	-.033	-,297*	,306*	,467**	-.014	.261	.015	.082	.202	-.030	.014	-.019	-.072	.158
Vacation	.097	.226	1	-.086	.182	.284	.204	-.069	.287	.004	-.059	.149	.138	-.180	.099	-.131	.168	.131	-.081	.044
Culture/ Atmo.	-.052	-.054	-.203	1	.108	.181	.090	-.054	-.018	.091	-.043	.241	-.163	.022	.115	.111	-.016	-.018	.233	-.160
Capacity	.083	.010	,391**	.087	1	-.056	.003	-.180	.202	.099	-.267	.122	.071	.264	.030	.233	.000	.100	.200	.262
Resources	.194	-.097	,364*	-,331*	,351*	1	.250	-.158	,296*	-.038	.246	.278	.255	.111	,403**	.182	.277	-.036	.099	-.045
Leadership	.239	-.082	-.147	,346*	-.143	-.178	1	-.105	.139	-.117	.142	.001	.098	-.199	.072	.087	.115	.011	-.200	.155
Performance	-.106	-.084	-.060	-.001	-.014	.115	-.016	1	-.137	-.193	.127	-.122	-.081	-.005	-.092	-.178	-.114	-,370*	-.037	-.153
Onboarding	.281	-.098	.107	-.069	.049	.087	.112	.075	1	.045	.123	.028	.282	-.133	.051	.191	.088	.210	-.088	.242
Work. Env.	.183	.093	.124	-,292*	-.047	.037	-.158	.054	-.035	1	-.052	.119	-.100	.082	-.099	.058	-.109	-.047	-.077	.221

Cooperation	.039	.200	.100	.097	.104	-.229	-.059	-.078	-.036	.021	1	-,363*	.079	-.047	.038	-.033	,337*	-.189	-.053	.085
Tech. Service	-.102	.077	.065	-.011	,376*	.268	.056	-.058	-.148	.075	-.205	1	-.001	.004	.111	.063	-.033	.090	.116	-.095
Administration	,333*	.120	.058	-.007	.051	.094	-.097	.003	.272	,361*	.167	-.232	1	.099	-.001	.252	-.183	.149	.047	.005
Work-Life	-.081	-.077	.180	.123	.113	.169	-.151	.054	.020	.052	.089	.044	.168	1	.093	.218	-.217	-.109	-.118	-.051
Supervision	.141	.033	.152	-.059	,354*	.187	-.034	.003	-.054	.245	.153	.116	.062	.051	1	.066	.171	-.079	.029	-.133
Salary	-.066	-.039	-.192	-.136	.198	.231	.073	-.189	-.006	.096	.034	.227	.172	.030	-.025	1	-.074	.025	-.091	,399**
Career Pers.	-.107	.014	.041	.046	-.196	-.116	-.134	.027	-,384**	.096	.230	-.165	-.155	-.124	.083	-.164	1	.035	-.025	.000
Work. flex.	,301*	-.019	,296*	-.153	.087	.151	-.052	.017	.218	.087	.021	-,323*	.104	.119	.033	-.107	.022	1	-.056	-.081
Coop. Nurses	.114	-.086	-.047	.093	-.032	-.026	.269	.176	-.215	-.094	-.028	-.037	.056	-.004	.220	.015	-.120	.032	1	.005
Work hours	-.085	.158	.178	-.116	,314*	.122	-.178	.044	-.074	-.237	,489**	.108	-.024	.084	-.076	,402**	-.023	-.052	-.026	1

* $p<.05$ (2-tailed)

** $p<.01$ (2-tailed)

Appendix 3: Suggestions concerning improvements with affiliated topics and frequencies of mentions in the interviews (multiple mentions only)

Suggestion for improvement	Number of persons proposing the improvement
Optimize employee appraisals: regular, structured, binding, more time, more importance, documentation (topic category: personnel management)	27
Making career prospects transparent and offering them, e.g., functional senior physician, senior physician positions (topic category: specialist retention)	23
Longer assignments - do not plug gaps and help out (topic category: specialist commitment)	19
Creating niches/specializations, e.g., outdoor areas, outpatient clinic (topic category: specialist retention)	19
Financial support for further training (topic category: specialist retention)	15
Continue rotation/target agreement discussions also for physicians, not only for residents (topic category: personnel management)	11
Improve tone and appreciation (topic category: specialist retention)	11
Offer language courses - German as a prerequisite (topic category: onboarding)	10
Salary increase (adapted to regional institutions) (topic category: specialist retention)	9
Exemption for further training (topic category: specialist retention)	9
Consider wishes for areas of application and activities (according to intensive WB) (topic category: specialist commitment)	9
Qualify managers and implement what they have learned (topic category: personnel management)	8
Creating a specialist curriculum (topic category: strengths/weaknesses)	8
Promoting a sense of togetherness and exchange, creating at home, e.g., arrangements and rooms for breaks (topic category: strengths/weaknesses)	8
Complete rosters earlier (topic category: specialist retention)	8
Flexibilization of working time/introduction of individual working time models, e.g., part-time, flextime) (topic category: specialist retention)	8
Optimize feedback culture, especially regular feedback, day-to-day feedback (topic category: personnel management)	7

Holiday planning more transparent (online calendar, exchange exchange), more binding (topic category: specialist retention)	7
Use of IT to bundle planning - professionalization (topic category: processes)	7
Permanent mentor for colleagues from abroad (topic category: onboarding)	7
Creating a better compatibility with social life (topic category: specialist retention)	5
Increase commitment, deadlines and feedback must also apply for superiors (topic category: personnel management)	5

Appendix 4: Results of discriminant analyses of frequencies of negative and positive statements to predict NPS groups (sceptical, neutral, promoting) concerning work, training, and intention to stay

Table A4.1: Discriminant analysis predicting NPS work by topics (number of negative and positive statements; absolute differences of function coefficients, Delta, marked according to amount)

	Test of equality of group means			Function coefficients NPS Work			Delta
	Wilks' Lambda	F[2,43]	Sig.	sceptical	neutral	promoting	
Quality of training neg	.978	.488	.617	37.257	40.207	24.077	32.261
Duty scheduling neg	.889	2.676	.080	22.184	16.222	18.461	11.925
Vacation scheduling neg	.916	1.971	.152	56.533	43.771	50.420	25.524
Culture / atmosphere neg	.992	.165	.849	-51.153	-20.666	-41.890	60.974
Personnel capacity neg	.957	.972	.386	-93.410	-67.623	-83.517	51.575
Resources / equipment neg	.773	6.312	.004	-93.904	-41.387	-82.337	105.035
Leadership neg	.958	.935	.401	66.266	21.838	43.815	88.855
Performance orientation neg	.986	.307	.737	123.823	88.156	110.779	71.333
Onboarding neg	.949	1.158	.324	45.720	18.062	30.333	55.316
Working environment neg	.950	1.133	.331	-161.981	-97.451	-176.045	157.190
Cooperation neg	.993	.154	.858	-54.885	-22.876	-44.319	64.019
Technical services neg	.954	1.046	.360	-15.175	-17.438	4.318	43.512
Administration neg	.917	1.943	.156	-36.745	-33.858	-21.509	30.472
Family / Work Life neg	.926	1.708	.193	310.500	160.925	244.900	299.149
Quality of supervision neg	.946	1.236	.301	-32.514	-4.846	-15.583	55.337
Salary neg	.901	2.370	.106	314.544	232.829	251.877	163.429
Career perspectives neg	.986	.294	.746	111.740	56.244	88.663	110.993
Flexible work schedules neg	.970	.672	.516	-149.333	-72.909	-99.217	152.848

Cooperation with nurses neg	.998	.034	.966	97.246	59.595	73.998	75.302
Working hours neg	.988	.251	.780	-216.479	-139.996	-176.958	152.964
Quality of training pos	.967	.728	.489	20.901	20.130	11.799	18.203
Duty scheduling pos	.988	.268	.766	155.384	94.664	115.027	121.440
Vacation scheduling pos	.938	1.420	.253	-164.338	-73.328	-141.490	182.021
Culture / atmosphere pos	.980	.444	.644	-104.370	-81.618	-83.286	45.504
Personnel capacity pos	.936	1.482	.239	-349.195	-131.531	-271.254	435.328
Resources / equipment pos	.994	.132	.876	31.515	-3.308	34.582	75.779
Leadership pos	.916	1.969	.152	32.442	29.201	34.569	10.736
Performance orientation pos	.977	.503	.608	-331.313	-206.656	-268.133	249.313
Onboarding pos	.889	2.687	.079	87.455	47.461	75.598	79.988
Working environment pos	.837	4.186	.022	101.306	63.762	118.113	108.703
Cooperation pos	.991	.204	.816	-373.217	-253.079	-299.877	240.275
Technical services pos	.993	.161	.852	-39.638	-19.518	-30.623	40.240
Administration pos	.952	1.092	.345	-147.025	-81.050	-113.008	131.950
Family / Work Life pos	.963	.820	.447	-108.013	-46.827	-49.686	122.372
Quality of supervision pos	.996	.076	.927	172.390	113.752	139.751	117.274
Salary pos	.958	.935	.401	-19.028	-83.479	-53.790	128.902
Career perspectives pos	.961	.877	.423	431.053	280.072	378.624	301.962
Flexible work schedules pos	.998	.043	.958	136.970	76.583	110.044	120.773
Cooperation with nurses pos	.982	.397	.675	130.276	75.736	115.312	109.080
Working hours pos	.994	.120	.887	-39.975	-7.752	-36.755	64.446
100% or original cases correctly classified				-174.161	-115.370	-139.324	

Table A4.2: Discriminant analysis predicting NPS <u>training</u> by topics (number of negative and positive statements; absolute differences of function coefficients, Delta, marked according to amount)

	Test of equality of group means			Function coefficients NPS Work			Delta
	Wilks' Lambda	F[2,43]	Sig.	sceptical	neutral	promoting	
Quality of training neg	,937	1,450	,246	80.340	50.278	50.287	60.125
Duty scheduling neg	,987	,277	,759	46.885	25.891	26.340	41.988
Vacation scheduling neg	,942	1,327	,276	73.568	57.124	53.562	40.012
Culture / atmosphere neg	,993	,141	,869	-51.759	-29.702	-29.196	45.125
Personnel capacity neg	,881	2,898	,066	-145.324	-96.222	-93.914	102.818
Resources / equipment neg	,903	2,299	,113	-68.216	-49.683	-47.862	40.710
Leadership neg	,989	,244	,784	33.361	12.500	16.898	41.723
Performance orientation neg	,999	,028	,972	186.976	126.833	121.877	130.198
Onboarding neg	,994	,121	,886	22.757	12.863	13.878	19.788
Working environment neg	,979	,465	,631	-208.505	-152.126	-146.614	123.783
Cooperation neg	,997	,075	,928	-74.541	-36.891	-38.289	75.301
Technical services neg	,983	,363	,697	-48.791	-24.148	-22.188	53.206
Administration neg	,992	,174	,841	-86.605	-49.706	-48.276	76.657
Family / Work Life neg	,902	2,325	,110	350.874	213.515	209.486	282.776
Quality of supervision neg	,868	3,277	,047	-55.565	-15.697	-17.353	79.735
Salary neg	,868	3,272	,048	468.645	321.753	302.667	331.957
Career perspectives neg	,980	,448	,642	107.956	68.646	67.556	80.800
Flexible work schedules neg	,977	,513	,603	-209.403	-108.402	-106.260	206.287
Cooperation with nurses neg	1,000	,008	,992	120.247	75.137	74.086	92.323
Working hours neg	,997	,074	,929	-288.876	-195.096	-184.067	209.619
Quality of training pos	,980	,439	,648	42.243	27.270	25.399	33.687

Duty scheduling pos	,942	1,323	,277	216.929	132.983	127.097	179.664
Vacation scheduling pos	,962	,856	,432	-96.114	-85.504	-75.894	40.440
Culture / atmosphere pos	,966	,768	,470	-179.561	-117.007	-111.849	135.424
Personnel capacity pos	,970	,671	,517	-380.248	-195.801	-196.952	368.894
Resources / equipment pos	,962	,839	,439	40.693	10.743	14.071	59.900
Leadership pos	,941	1,349	,270	38.410	35.212	33.834	9.153
Performance orientation pos	,965	,784	,463	-477.794	-290.062	-286.588	382.411
Onboarding pos	,941	1,349	,270	120.416	76.362	71.794	97.243
Working environment pos	,920	1,872	,166	107.165	86.641	86.910	41.048
Cooperation pos	,949	1,164	,322	-566.579	-358.365	-346.845	439.469
Technical services pos	,989	,231	,794	-94.079	-38.922	-43.434	110.313
Administration pos	,984	,343	,711	-183.888	-121.080	-109.792	148.193
Family / Work Life pos	,980	,429	,654	-105.673	-43.986	-46.481	123.373
Quality of supervision pos	,974	,567	,571	198.765	143.718	135.545	126.441
Salary pos	,937	1,445	,247	-141.622	-104.742	-115.998	73.760
Career perspectives pos	,940	1,378	,263	657.578	404.860	404.049	507.057
Flexible work schedules pos	,968	,708	,498	128.314	90.318	86.948	82.732
Cooperation with nurses pos	,983	,364	,697	140.093	100.481	95.755	88.675
Working hours pos	,970	,671	,517	59.492	15.624	18.163	87.736
95.7% or original cases correctly classified				-265.861	-146.466	-131.962	

Table A4.3: Discriminant analysis predicting NPS intention to stay by topics (number of negative and positive statements; absolute differences of function coefficients, Delta, marked according to amount)

	Test of equality of group means			Function coefficients NPS Work			Delta
	Wilks' Lambda	F[2,43]	Sig.	sceptical	neutral	promoting	
Quality of training neg	.987	.292	.748	28.781	40.519	30.124	23.477
Duty scheduling neg	.814	4.899	.012	15.245	16.710	20.233	9.976
Vacation scheduling neg	.959	.912	.409	33.397	45.546	24.900	41.291
Culture / atmosphere neg	.968	.708	.498	-7.562	-16.182	6.960	46.284
Personnel capacity neg	.890	2.664	.081	-55.562	-71.553	-52.002	39.103
Resources / equipment neg	.927	1.698	.195	-36.146	-41.699	-44.992	17.692
Leadership neg	.951	1.110	.339	10.862	-7.049	1.810	35.822
Performance orientation neg	.984	.350	.706	75.504	87.440	71.905	31.071
Onboarding neg	.967	.742	.482	4.122	8.051	-5.936	27.975
Working environment neg	.911	2.088	.136	-103.111	-106.304	-94.841	22.927
Cooperation neg	.979	.464	.632	-23.617	-13.759	-34.775	42.030
Technical services neg	.988	.253	.778	-2.590	-.091	7.473	20.125
Administration neg	.875	3.084	.056	-10.725	-34.100	7.807	83.814
Family / Work Life neg	.995	.111	.895	110.000	142.759	105.078	75.361
Quality of supervision neg	.964	.803	.455	10.823	4.308	20.770	32.924
Salary neg	.854	3.684	.033	153.915	201.404	78.374	246.061
Career perspectives neg	.969	.695	.505	36.305	41.390	22.813	37.154
Flexible work schedules neg	.975	.546	.583	1.127	-43.830	87.238	262.136
Cooperation with nurses neg	.968	.707	.499	38.640	60.096	37.894	44.404
Working hours neg	.926	1.712	.193	-85.728	-131.581	-38.549	186.063
Quality of training pos	.921	1.856	.169	8.496	13.452	-4.344	35.593
Duty scheduling pos	.966	.758	.475	43.166	61.760	-18.534	160.589

Vacation scheduling pos	.996	.077	.926	-44.396	-54.991	-8.220	93.542
Culture / atmosphere pos	.980	.431	.653	-48.794	-72.527	-12.388	120.277
Personnel capacity pos	.982	.389	.680	-54.364	-111.932	-15.758	192.347
Resources / equipment pos	.946	1.226	.304	4.307	-3.584	12.503	32.173
Leadership pos	.975	.546	.583	30.372	39.326	37.897	17.907
Performance orientation pos	.971	.634	.535	-106.034	-181.076	8.016	378.185
Onboarding pos	.974	.574	.568	35.728	55.989	40.009	40.521
Working environment pos	.870	3.216	.050	79.661	94.694	107.683	56.044
Cooperation pos	.970	.668	.518	-158.617	-218.296	-66.965	302.661
Technical services pos	.896	2.485	.095	-18.857	4.300	-11.582	46.313
Administration pos	.972	.619	.543	-40.626	-70.662	-9.694	121.937
Family / Work Life pos	.904	2.276	.115	-6.979	15.305	25.578	65.112
Quality of supervision pos	.925	1.747	.187	71.206	125.976	54.214	143.525
Salary pos	.965	.770	.469	-123.655	-66.562	-128.074	123.023
Career perspectives pos	.982	.393	.677	207.784	268.358	142.409	251.899
Flexible work schedules pos	.976	.532	.591	49.811	60.608	28.457	64.301
Cooperation with nurses pos	.969	.698	.503	63.858	77.544	66.879	27.371
Working hours pos	.989	.244	.784	2.062	-3.215	21.010	48.450
100% or original cases correctly classified				-113.550	-130.928	-207.983	

Appendix 5: Item intercorrelation of polarity satisfaction scale

No		1	2	3	4	5	6	7	8	9	10	11	12	13
1	appreciation and support by colleagues													
2	appreciation and support by superiors	.39**												
3	regard of personal goals	.26**	.51**											
4	reliability promises kept	0.02	0.06	0.19										
5	foresighted planning	0.09	.21*	.36**	0.09									
6	sufficient information in time	.24**	.40**	.41**	0.11	.51**								
7	decisions comprehensible	0.15	.47**	.51**	0.07	.25**	.55**							
8	self-directed work	.19*	.22*	.33**	-0.03	0.03	.35**	.23*						
9	challenged but not overwhelmed	0.02	-0.01	0.08	-0.01	0.16	-0.06	-0.04	-0.09					
10	useful purposeful work	0.10	.20*	.27**	0.08	.20*	.23*	0.16	0.12	0.06				
11	good working environment	0.12	.21*	.33**	0.03	0.16	.34**	.31**	.19*	0.04	0.16			
12	fair salary	0.13	0.14	.29**	.19*	.25**	0.11	0.18	-0.09	0.09	.21*	.22*		
13	good career perspectives	0.16	.40**	.45**	0.04	0.18	.38**	.37**	.26**	0.06	.28**	.36**	.28**	
14	working hours manageable	0.12	0.17	.21*	0.08	.33**	.30**	.19*	0.11	0.12	.24*	.22*	.30**	0.12

* p<.05 (2-tailed), ** p<.01 (2-tailed)

Appendix 6 – Table A6.1: Discriminant analysis predicting number of promoters concerning work, training, and intention (from 0=none to 3=all three) by items of the PhysicianPlus satisfaction scale (p<.10)

	Test of equality of group means					Fisher's function coefficients			
Items	Mean	SD	Wilks' Lambda	F[3,106]	Sig.	promotes none	promotes 1 NPS	promotes 2 NPS	promotes 3 NPS
appreciation and support by colleagues	1.85	0.95	0.97	1.30	0.28	1.539	.892	1.484	1.352
appreciation and support by superiors	2.09	1.06	0.94	2.17	0.10	-.099	.674	-.303	.967
regard of personal goals	2.55	1.40	0.89	4.56	0.00	-.575	-.982	.089	-.790
reliability promises kept	2.99	5.56	0.96	1.61	0.19	.010	-.013	.143	.008
foresighted planning	3.06	1.80	0.91	3.52	0.02	.755	.589	.363	.421
sufficient information in time	3.05	1.49	0.84	6.80	0.00	-.368	-.460	-.185	-.637
decisions comprehensible	2.68	1.42	0.82	7.91	0.00	1.229	.887	.437	.351
self-directed work	2.28	1.20	0.97	1.06	0.37	.929	1.199	.972	1.008
challenge but not overwhelmed	2.51	5.51	0.99	0.37	0.77	.055	.022	.036	.015
useful purposeful work	1.54	0.94	0.94	2.43	0.07	.921	.533	.819	.370
good working environment	3.78	1.62	0.86	5.74	0.00	1.150	1.015	.730	.685
fair salary	2.68	1.68	0.95	1.70	0.17	-.231	.437	-.197	.230
good career perspectives	2.45	1.40	0.78	10.26	0.00	1.143	.602	.056	.225
working hours manageable	3.43	1.19	0.95	1.90	0.13	1.875	1.567	1.747	1.509
100% correct classified, 100% variance explained						-13.763	-10.397	-8.879	-7.431

Table A6.2: Means and standard deviations (SD) of items of the PhysicianPlus job satisfaction scale for persons promoting (rating 9-10) none, one, two, or three NPS aspects work, training, and intention to stay.

	promotes none			promotes one (work, training, or intention to stay)			promotes two (work, training, or intention to stay)			promotes three (work, training, or intention to stay)		
	Mean	SD	n	Mean	SD	n	Mean	SD	n	Mean	SD	n
appreciation and support by colleages	1.99	0.96	66	1.56	0.65	25	1.75	1.13	13	1.80	1.24	8
appreciation and support by superiors	2.25	1.11	66	2.06	0.97	25	1.48	0.68	13	1.88	1.18	8
regard of personal goals	2.90	1.31	66	2.06	1.33	25	2.40	1.68	13	1.43	0.90	8
reliability promises kept	2.89	1.47	66	2.16	1.33	25	5.95	16.07	13	1.61	0.97	8
foresighted planning	3.47	1.80	66	2.71	1.71	25	2.45	1.44	13	1.80	1.76	8
sufficient information in time	3.50	1.45	66	2.64	1.32	25	2.47	1.41	13	1.58	0.78	8
decisions comprehensible	3.12	1.35	66	2.42	1.43	25	1.75	1.11	13	1.31	0.51	8
self directed work	2.43	1.26	66	2.16	1.04	25	2.12	1.31	13	1.73	0.93	8
challenge but not overwhelmed	2.96	7.12	66	1.80	0.73	25	2.08	1.33	13	1.65	0.77	8
useful purposeful work	1.72	0.95	66	1.32	0.77	25	1.38	1.18	13	0.98	0.45	8
good working environment	4.20	1.45	66	3.60	1.59	25	2.82	1.67	13	2.40	1.73	8
fair salary	2.69	1.72	66	3.18	1.50	25	2.01	1.59	13	2.18	1.84	8
good career perspectives	2.94	1.42	66	2.18	1.09	25	1.22	0.58	13	1.28	0.81	8
working hours manageable	3.63	1.12	66	3.24	1.19	25	3.18	1.21	13	2.78	1.47	8

Appendix 7: Study Guide Rep Grid

Table A7.1: Principal components and factor loadings for the first two components as for work promotors and sceptics

	Eigenvalues work Promoters			Eigenvalues work Sceptics		
	Eigenvalue %	Variance	Cumulative %	Eigenvalue %	Variance	Cumulative %
PC_1	8525.62	63.28	63.28	4877.60	56.38	56.83
PC_2	1525.79	11.33	74.61	1201.63	14.00	70.83

	Factor loadings Promoters		Factor loadings Sceptics	
	PC_1	PC_2	PC_1	PC_2
Physicians	42.92	1.37	38.32	-1.72
Nurses	37.06	21.09	39.36	-9.17
Clinic today	25.97	-3.87	6.60	4.47
Clinic in 5 years	9.12	-26.43	-2.21	20.75
Hospital Admin.	-45.25	13.39	-26.89	15.66
Clinic Admin.	0.84	-3.58	-12.33	8.27
Hospital today	-33.14	8.28	-18.40	12.34
Hospital in 5 years	-37.50	-10.24	-24.46	4.65

Table A7.2: Principal components and factor loadings for the first two components as for staff members with low vs. high intention-to-stay

	Eigenvalues high intention to stay			Eigenvalues low intention to stay		
	Eigenvalue %	Variance	Cumulative %	Eigenvalue %	Variance	Cumulative %
PC_1	4028.18	62.04	62.04	12967.54	60.28	60.28
PC_2	984.45	15.16	77.21	2755.46	12.81	73.09

	Factor loadings high intention		Factor loadings low intention	
	PC_1	PC_2	PC_1	PC_2
Physicians	30.83	-3.52	57.09	-4.62
Nurses	26.39	-15.00	57.60	-25.01
Clinic today	15.69	4.70	22.22	15.13
Clinic in 5 years	7.19	22.99	-0.96	27.13
Hospital Admin.	-30.63	-9.19	-49.31	-13.34
Clinic Admin.	-1.69	-0.67	-4.74	23.98
Hospital today	-23.86	-7.12	-34.31	-19.39
Hospital in 5 years	-23.93	7.81	-47.59	-3.88
